# ComEA Is Essential for the Transfer of External DNA into the Periplasm in Naturally Transformable *Vibrio cholerae* Cells

**DOI:** 10.1371/journal.pgen.1004066

**Published:** 2014-01-02

**Authors:** Patrick Seitz, Hassan Pezeshgi Modarres, Sandrine Borgeaud, Roman D. Bulushev, Lorenz J. Steinbock, Aleksandra Radenovic, Matteo Dal Peraro, Melanie Blokesch

**Affiliations:** 1 Laboratory of Molecular Microbiology, Global Health Institute, School of Life Sciences, Ecole Polytechnique Fédérale de Lausanne (EPFL), Lausanne, Switzerland; 2 Laboratory for Biomolecular Modeling, Institute of Bioengineering, School of Life Sciences, Ecole Polytechnique Fédérale de Lausanne (EPFL), Lausanne, Switzerland; 3 Swiss Institute of Bioinformatics (SIB), Lausanne, Switzerland; 4 Laboratory of Nanoscale Biology, Institute of Bioengineering, School of Engineering, Ecole Polytechnique Fédérale de Lausanne (EPFL), Lausanne, Switzerland; Institute of Molecular and Cell Biology (IMCB), A*STAR, Singapore

## Abstract

The DNA uptake of naturally competent bacteria has been attributed to the action of DNA uptake machineries resembling type IV pilus complexes. However, the protein(s) for pulling the DNA across the outer membrane of Gram-negative bacteria remain speculative. Here we show that the competence protein ComEA binds incoming DNA in the periplasm of naturally competent *Vibrio cholerae* cells thereby promoting DNA uptake, possibly through ratcheting and entropic forces associated with ComEA binding. Using comparative modeling and molecular simulations, we projected the 3D structure and DNA-binding site of ComEA. These *in silico* predictions, combined with *in vivo* and *in vitro* validations of wild-type and site-directed modified variants of ComEA, suggested that ComEA is not solely a DNA receptor protein but plays a direct role in the DNA uptake process. Furthermore, we uncovered that ComEA homologs of other bacteria (both Gram-positive and Gram-negative) efficiently compensated for the absence of ComEA in *V. cholerae*, suggesting that the contribution of ComEA in the DNA uptake process might be conserved among naturally competent bacteria.

## Author Summary

Horizontal gene transfer (HGT) plays a key role in transferring genetic information from one organism to another. Natural competence for transformation is one of three modes of HGT used by bacteria to promote the uptake of free DNA from the surrounding. The human pathogen *Vibrio cholerae* enters such a competence state upon growth on chitinous surfaces, which represent its natural niche in the aquatic environment. Whereas we have gained a reasonable understanding on how the competence phenotype is regulated in *V. cholerae* we are only at the beginning of deciphering the mechanistic aspects of the DNA uptake process. In this study, we characterize the competence protein ComEA. We show that ComEA is transported into the periplasm of *V. cholerae* and that it is required for the uptake of DNA across the outer membrane. We demonstrate that ComEA aggregates around incoming DNA *in vivo* and that the binding of DNA is dependent on specific residues within a conserved helix-hairpin-helix motif. We propose a model indicating that the DNA uptake process across the outer membrane might be driven through ratcheting and entropic forces associated with ComEA binding.

## Introduction

Recombination between the bacterial chromosome and DNA fragments that enter the cell through horizontal gene transfer (HGT) either replace damaged or mutated alleles with the original alleles, thereby repairing the gene, or transfer mutated alleles or new genes to naïve strains. Thus, HGT plays a key role in transferring genetic information from one bacterium to another and maintaining the balance between genome maintenance and evolution. Natural competence for transformation is one of three modes of HGT in bacteria and promotes the uptake of free DNA from the environment (for recent reviews see [1]–[6]).

Many naturally transformable bacteria have been described [7], including the pathogenic bacterium *Vibrio cholerae* [6], [8]. The physiological state of natural competence of this Gram-negative bacterium is associated with its primary niche, the aquatic environment. Within this habitat, *V. cholerae* attaches to the exoskeleton of zooplankton or zooplankton molts [9]. Those exoskeletons comprise the polymer chitin, which is the natural inducer of competence in *V. cholerae* [6], [8], [10]. Whereas the regulatory network driving competence has been well investigated (reviewed by Seitz and Blokesch [6]), so far very little is known about the DNA uptake complex of *V. cholerae* [11]. With respect to the DNA uptake machinery of naturally transformable bacteria it has been suggested that a (pseudo-)pilus [1], [2], similar to type IV pili (Tfp) [12], represents a core element of the DNA import machinery. However, it is still unclear how the proteins interact to pull the transforming DNA through the cell envelope [3]. A proposed mechanism for DNA uptake involves repeating cycles of pilus extension and retraction [1], [2], [4], [13] although recent review articles suggested that other competence proteins, such as ComEA, might be involved in pulling the DNA into the cell [4], [14] (though without experimental evidence). The present study reinforces those ideas and shows that ComEA is a prerequisite for DNA uptake in naturally competent *V. cholerae*. Furthermore, based on an earlier study on DNA ejection from bacteriophages [15] we propose a model suggesting that the DNA translocation across the outer membrane is possibly accomplished by ratcheting and entropic forces associated with the binding of ComEA to the incoming DNA.

Currently, the majority of studies on the cellular localization of competence proteins were performed on Gram-positive bacteria [16]–[19], whereas far less is known about competence protein localization in Gram-negative bacteria. We recently identified the minimal competence gene set of *V. cholerae* and provided first insight into the DNA uptake machinery of this organism [11]. Notably, through the analysis of knockout strains lacking specific components of the DNA uptake complex we demonstrated that natural transformation still occurred in the absence of the proteins involved in the Tfp structure and biosynthesis though at very low frequencies. Such rare transformants were never detectable for *comEA* ^−^ strains [11], suggesting that ComEA plays an important role in the DNA uptake process, the focus of this work.

In studies on *B. subtilis* and *S. pneumoniae* it was reported that binding of transforming DNA to those Gram-positive cells is at least partially mediated by ComEA and that ComEA is “absolutely required” for DNA uptake and transformation [20]–[22]. Likewise, ComE (ComEA homolog)-negative strains of *Neisseria gonorrhoeae* [23] and *V. cholerae* [8], [24] were severely or completely impaired for natural transformability, indicating that ComEA might also play an important role in Gram-negative bacteria. A recent study by Lo Scrudato and Blokesch indicated that *comEA* and the gene encoding the inner membrane transporter *comEC* were differentially regulated from the Tfp-like components of the DNA uptake machinery [25], [26], which, together with our study on the DNA uptake machinery, suggest that DNA transport might be a multi-step process in *V. cholerae* (as previously proposed for *Helicobacter pylori* [14], [27], which does not contain a *bona fide* Tfp-based DNA uptake machinery). Here, we show that the Tfp-like elements of the DNA uptake machinery of *V. cholerae* are not sufficient to translocate DNA across the outer membrane and that the competence protein ComEA plays an essential role in this process.

## Results and Discussion

### ComEA localizes to the periplasm in naturally competent *V. cholerae* cells

**Figure 1:**
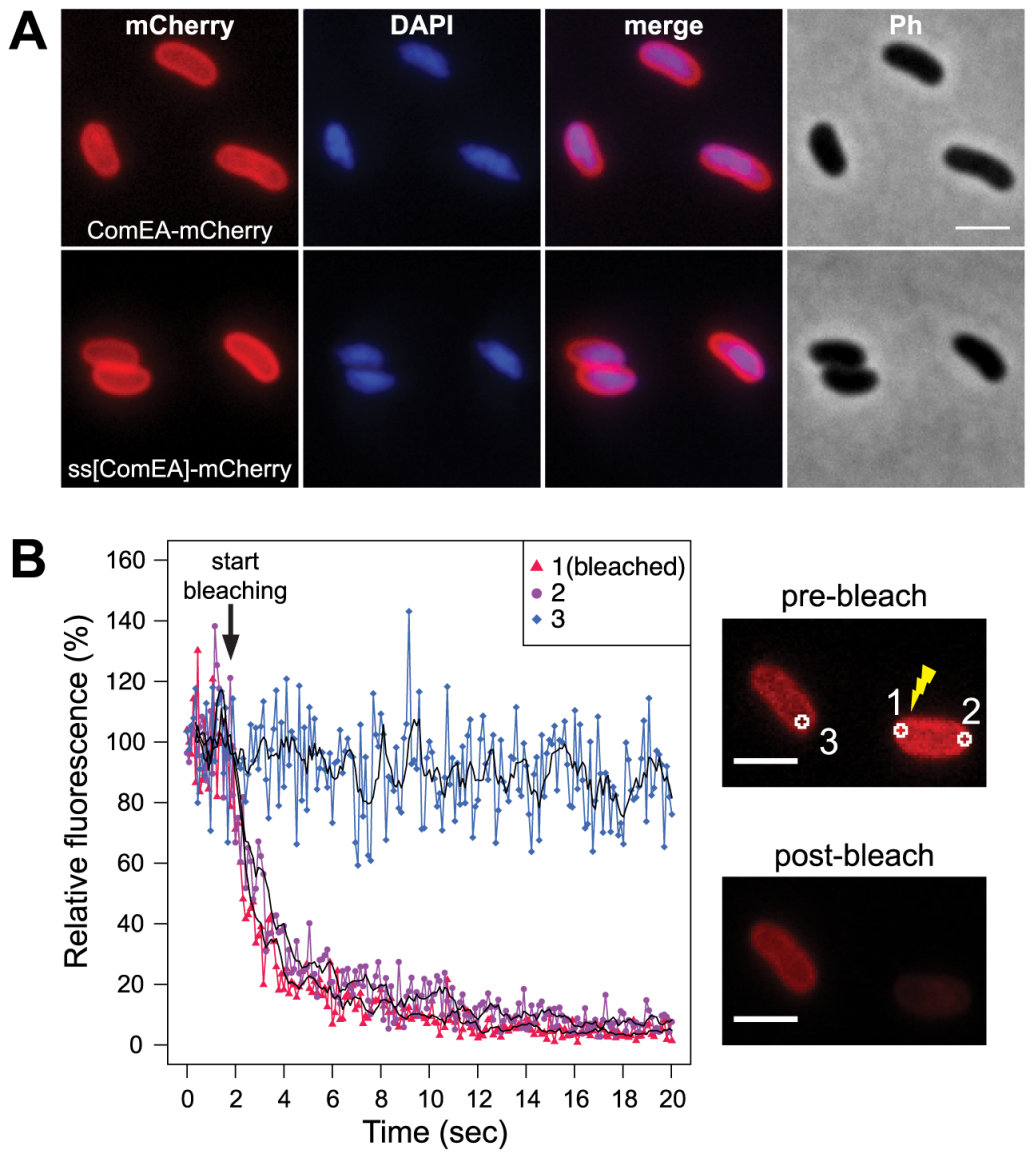
Localization of the ComEA protein in naturally competent *V. cholerae* cells. (A) Expression and distribution of ComEA-mCherry (upper row) or signal sequence[ComEA] (amino acids 1–25)-mCherry fusion proteins (lower row) within competent *V. cholerae* cells. Fluorescent signals for mCherry or DAPI-stained genomic DNA were visualized and compared with each other (merge) and the corresponding phase contrast image (Ph). (B) Representative fluorescence loss in photobleaching (FLIP) experiment to demonstrate the degree of mobility of ComEA-mCherry in live bacteria. Bleaching of the region-of-interest (ROI) 1 (indicated as 1 in the images on the right) was initiated after the acquisition of 20 frames and repeated after every frame. The fluorescence intensities of ROIs 1–3 were measured for a total of 20 sec and normalized to the average fluorescence intensity of the first 10 frames. The moving averages (period n = 5) are indicated with black lines. The average fluorescence intensity projections before (pre-bleach) and after bleaching (post-bleach) are shown on the right. Scale bars, 2 µm.

In a previous study by Chen and Gotschlich the authors predicted a 19-residues signal sequence for sec-dependent transport of the ComEA-homolog of *Neisseria gonorrhoeae* (ComE) into the periplasm [23]. Such a predictable signal sequence (amino acid residues 1–25) is also present in ComEA of *V. cholerae*. To experimentally address the localization of the ComEA protein we aimed at visualizing it *in vivo* by constructing a functional translational fusion between ComEA and mCherry. Using this construct we observed a uniform localization pattern of ComEA (Fig. 1A), which is consistent with the presence of such an N-terminal signal sequence and the transport of ComEA to the periplasm. To validate this microscopical observation, we generated a translational fusion between *comEA* and the gene encoding beta-lactamase (*bla*; without the region encoding the signal sequence), which replaced the wild-type *comEA* allele on the *V. cholerae* large chromosome. The resulting strain retained natural transformability at a frequency of 2.5×10^−5^±3.0×10^−5^ compared with 7.9×10^−5^±2.5×10^−5^ for the parental wild-type strain (average of four biological replicates ± SD) indicating the functionality of the fusion construct. Most importantly, the construct conferred full resistance to ampicillin, which provides further evidence for the periplasmic localization of ComEA-bla as beta-lactamase can only exert activity against beta-lactam antibiotics in the periplasm of Gram-negative bacteria (Fig. S1).

Next, we aimed to investigate whether the ComEA protein is motile within the periplasm. To this extent we used a fluorescence loss in photobleaching (FLIP; Fig. 1B) approach because photobleaching can reveal protein dynamics in live cells [28]. In contrast to fluorescence recovery after photobleaching (FRAP), where fluorescent proteins within a small area of the cell are bleached and the back-diffusion of the surrounding non-bleached proteins into this region is recorded, FLIP consist of repetitive bleaching of the same region (e.g. region of interest 1 in Fig. 1B), thereby preventing fluorescence recovery in that region. Moreover, any mobile protein from elsewhere in the same compartment (e.g. region of interest 2 in Fig. 1B) will also enter this continuously photo-bleached area, eventually resulting in a complete loss of fluorescence in the compartment. In contrast, any not connected compartment will be spared from bleaching (e.g. region of interest 3 in Fig. 1B). Therefore, FLIP is often used to reveal the mobility of proteins within certain compartments of the cell [29], which is what we were aiming for. Indeed, our FLIP experiments indicated that ComEA was highly motile within the periplasm (Fig. 1B). Likewise, a translational fusion between the signal sequence of ComEA (amino acid residues 1–25; ss[ComEA]) alone and mCherry resulted in a similar localization (Fig. 1A) and mobility pattern (Fig. S2). This uniform localization pattern differed from that obtained from previous studies on *B. subtilis*, where Hahn *et al.* used immunofluorescence microscopy to show that ComEA localizes in a non-uniform punctate manner [16]. Kaufenstein *et al.* confirmed those data and concluded that the distinct assemblies of ComEA were mobile [19].

### ComEA binds to DNA *in vivo*

**Figure 2:**
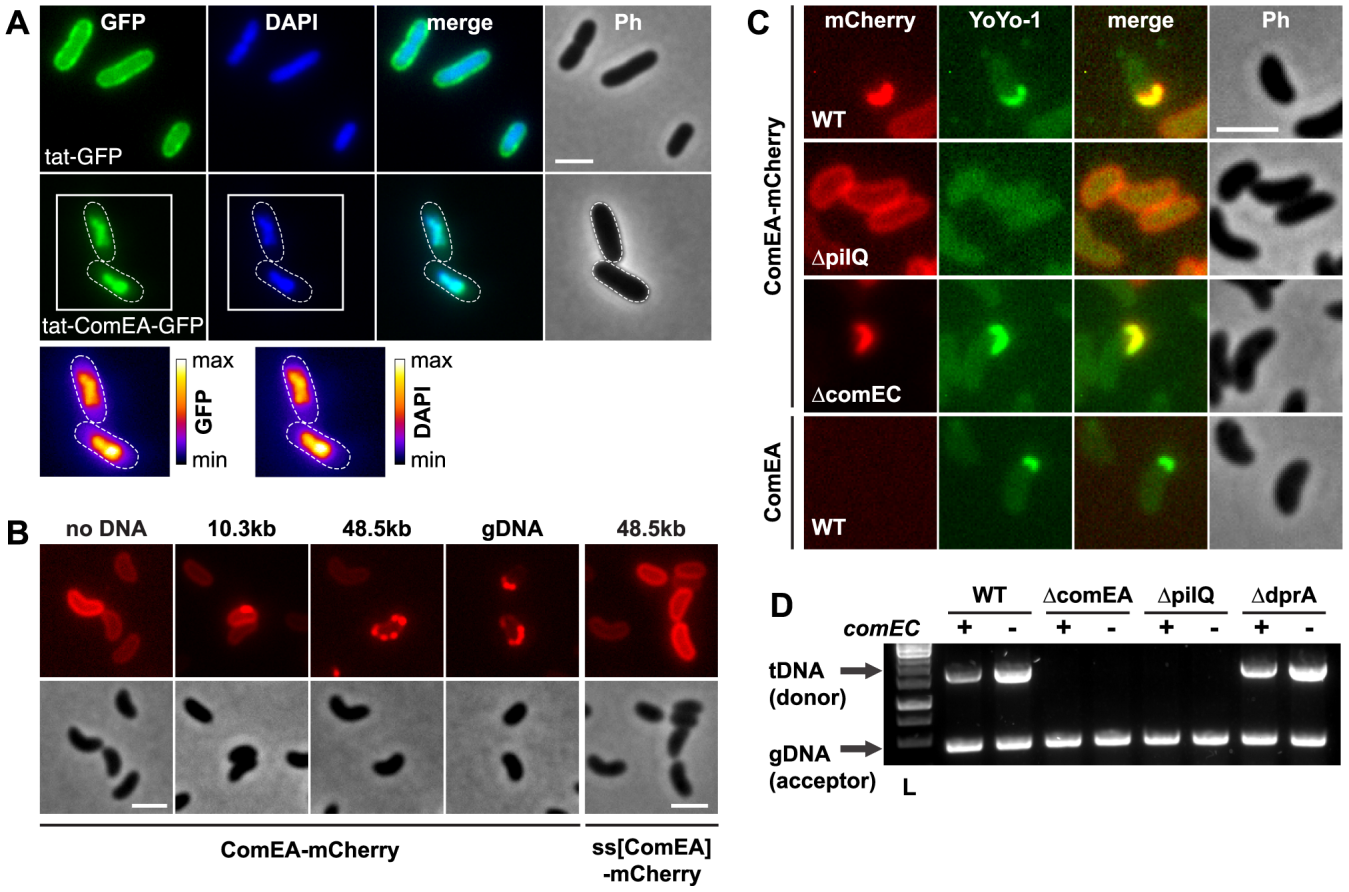
ComEA binds to DNA *in vivo*. (A) Plasmid-encoded *gfp* (tat-GFP) or *comEA-gfp* (tat-ComEA-GFP), both preceded by a tat-signal sequence, were expressed in *E. coli*. The images shown correspond to the GFP channel, DAPI channel (to visualize DAPI-stained DNA), merged fluorescent images (merge), and phase contrast (Ph). The cells are outlined with dashed lines for tat-ComEA-GFP. Heat-maps showing the fluorescence intensities of the GFP and DAPI signals are depicted for the *tat-comEA-gfp* expressing cells below the images. (B) ComEA-mCherry aggregation and foci formation after the addition of external DNA. Competence-induced cells without (no DNA) or with external DNA were imaged in the red (mCherry; upper row) or the phase contrast channel (lower row) to visualize ComEA-mCherry localization. The DNA fragments differed in lengths (PCR fragment, 10.3 kb; λDNA, 48.5 kb; gDNA, various lengths). Transforming DNA did not lead to foci formation of periplasmic mCherry alone (preceded by the ComEA signal sequence; ss[ComEA]-mCherry). (C) Colocalization (merged image) of ComEA-mCherry (red channel) and YoYo-1-stained transforming DNA (green channel). The outline of the cells is shown in the phase contrast image (Ph). Scale bars in all images, 2 µm. (D) DNA uptake requires ComEA. DNA uptake of competent *V. cholerae* cells was tested using a whole-cell duplex PCR assay. All mutant strains were tested in a *comEC* positive (+) and negative (−) background. The lower PCR fragments indicate acceptor strain DNA (gDNA, acceptor); the upper band indicates internalized transforming DNA (tDNA). L, ladder.

Studies using purified tagged ComEA/ComE homologs demonstrated that the protein binds DNA *in vitro*; thus, ComEA was considered as a DNA receptor protein [21], [23], [30], [31]. DNA binding could be attributed to a conserved helix-hairpin-helix (HhH) motif [32]. Notably and in contrast to helix-turn-helix or helix-loop-helix motifs, which are widespread in proteins that interact with DNA in a sequence-dependent manner, HhH motifs bind DNA in a non-sequence-specific manner. Such binding is based on hydrogen bonding between the protein and the DNA phosphate groups [32] and HhH motifs have been described in various protein classes, including DNA polymerases, DNA ligases or DNA glycosylases [32], [33]. However, the *in vivo* binding of DNA through ComEA has never been demonstrated. We genetically engineered a fusion protein between ComEA and GFP, which was transported across the inner membrane via the Tat-transport machinery in a folded state (as GFP is improperly folded when translocated to the periplasm in a sec pathway dependent manner [34]). Interestingly, the protein failed to translocate in *Escherichia coli*; instead, ComEA was tightly bound to the bacterial chromosome, which appeared as a highly compacted structure (Fig. 2A). The increased protein expression levels resulted in cell death, indicating that the strong binding of ComEA to the DNA *in vivo* interfered with cellular processes. Due to this lack of translocation of ComEA-GFP into the periplasm and the *in vivo* binding to the chromosome we conducted further experiments using the ComEA-mCherry fusion despite the lower signal intensity of mCherry compared with GFP.

### ComEA is required for uptake of transforming DNA into the periplasm

To investigate the function of ComEA *in vivo*, we excluded artifacts caused by artificial (over-)expression as those have been recognized as having detrimental effects on subcellular localization [35]. Thus, all *V. cholerae* strains used in these experiments were generated through the substitution of chromosomal *comEA* with diverse *comEA-mCherry* alleles. In these strains, the expression of *comEA-mCherry* was driven through its native promoter and consequently co-regulated with other competence genes. The functionality of the chromosomally encoded ComEA fusion protein was confirmed using a transformation assay, and the chromosomally-encoded fusion protein was uniformly localized within the periplasm (Fig. 2B and Fig. S3). Importantly, the addition of external transforming DNA (tDNA) led to the formation of distinctive ComEA-mCherry foci (Fig. 2B). The size and numbers of these protein aggregates was dependent on the length of the supplemented tDNA. Periplasmic mCherry alone did not aggregate (ss[ComEA]-mCherry; Fig. 2B). A similar relocalization pattern after the addition of external DNA was also observed when the cells were grown on chitin surfaces mimicking the natural reservoir *V. cholerae* (Fig. S4). This observation suggested that ComEA binds transforming DNA in the periplasm thereby potentially contributing to DNA translocation across the outer membrane.

To test this hypothesis, we repeated the experiments using YoYo-1-labeled DNA. Indeed, a perfect colocalization pattern was observed when the fluorescent signals of ComEA-mCherry and DNA were compared (Fig. 2C). Foci formation through ComEA and colocalization with YoYo-1-labeled DNA were absent in a strain lacking the outer membrane pore PilQ [2], [8], [11], whereas the absence of the inner membrane transporter ComEC did not interfere with ComEA-DNA colocalization (Fig. 2C). Similar foci formation of YoYo-1-labeled DNA was also observed in a strain carrying wild-type ComEA, excluding a translational artifact resulting from the mCherry-fusion (Fig. 2C). Notably, YoYo-1 foci were absent in a *comEA*-negative strain, which was also the case for a strain lacking the major Tfp subunit PilA (Fig. S5A).

Using a whole-cell duplex PCR-based DNA uptake assay [24], [11] that aims at detecting DNA strands, which have either entered the periplasm or have already reached the cytoplasm of the competent bacteria (thereby becoming resistant against externally applied DNase), we confirmed that tDNA (both unlabeled or YoYo-1-labeled) was undetectable in *comEA*-negative strains even though it was readily detectable in the wild-type strain and in *comEC* negative derivatives (Fig. 2D and Fig. S5).

Whereas the absence of YoYo-1 labeled DNA foci and PCR-amplifiable DNA in *comEA* negative strains is indicative of a failure to transport tDNA across the outer membrane, such results would also be consistent with ComEA's main function being to protect and stabilize incoming tDNA against potential nucleases. Indeed, two nucleases have been described for *V. cholerae*, Dns and Xds, which are solely responsible for extracellular nuclease activity in this organism [36]. Interestingly, Focareta and Manning demonstrated that even though Dns can be recovered from culture supernatants, it was also detectable in the periplasmic space of *V. cholerae* [37]. We recently confirmed the extracellular localization of Dns [38] but also its at least partial association with the bacterial cells (through western blot analysis; [25]). Moreover, Blokesch and Schoolnik showed that expression of *dns* has to be silenced in *V. cholerae* to allow natural transformation to occur at high cell density [26], [38]. Thus, to rule out the possibility that ComEA might protect incoming tDNA against either of those two nucleases we tested *dns*, *xds*, and *comEA* single, double, and triple mutants for natural transformation and the recovery of DNase resistant tDNA in whole cells (Fig. S6). Notably, the absence of *dns* resulted in higher transformability (Fig. S6A), consistent with an early study [38], and in the detection of increased amounts of DNase-resistant tDNA within the bacteria (Fig. S6B). However, no transformants or translocated tDNA were detectable if *comEA* was concomitantly absent (Fig. S6). We therefore conclude that ComEA's main role is not to protect incoming tDNA against degradation by the nucleases Xds or Dns, though we cannot exclude the presence of any other hitherto unidentified nuclease in the periplasm of *V. cholerae*. Instead, we suggest that translocation of tDNA across the outer membrane is not solely driven through Tfp-like elements of the DNA uptake machinery but also requires ComEA.

### *In silico* prediction and *in vivo* validation of a ComEA-DNA complex

**Figure 3:**
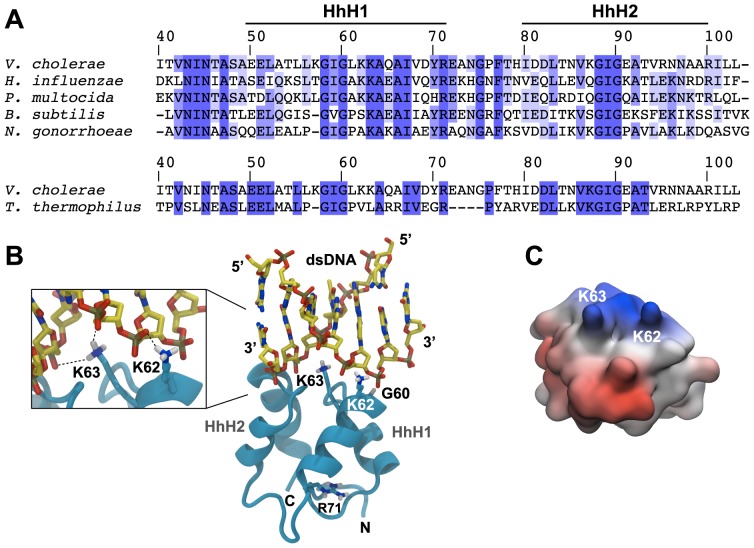
*In silico*-prediction of the ComEA-DNA complex. (A) Protein sequence alignment of the two helix-hairpin-helix motifs (HhH1-2) of ComEA/ComE homologs from the indicated organisms (using the ComEA residue numbering). The sequence conservation is shown in tones of blue (dark blue = highly conserved). (B) 3D model of ComEA and its predicted DNA binding mode based on comparative modeling using the ComEA-related protein of *T. thermophilus* HB8 (PDB ID: 2DUY) as a template (see also movie S1). The non-sequence-specific DNA backbone phosphate interactions with K62 and K63 are shown (inset). (C) The electrostatic potential at the molecular surface of ComEA is reported within a ±160 k_B_T/e range (negative values in red, positive values in blue).

To gain insights into the molecular mechanism through which ComEA binds dsDNA, we predicted the structure of ComEA and characterized the interactions of this protein with the transforming DNA. First, we used comparative modeling to create a 3D structure of ComEA using the X-ray structure of the ComEA-related protein HB8 from *Thermus thermophilus* (PDB ID: 2DUY, unpublished) as a template (Fig. 3, movie S1). Based on structural similarity with structures from the HhH family [39], we identified K62 and K63 as candidate residues for DNA binding interactions and could model the putative ComEA-DNA adduct (Fig. 3B). The electrostatic potential of the ComEA model is consistent with the identified DNA-binding region, showing positively charged regions corresponding to the lysine pair (Fig. 3C).

**Figure 4:**
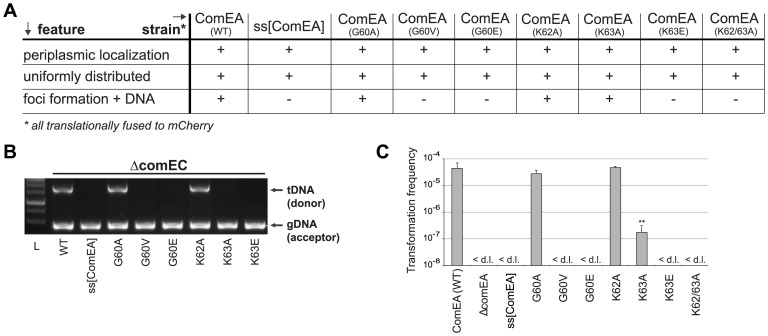
*In vivo* validation of ComEA-DNA interaction sites. Strains carrying *comEA-mCherry* or variants thereof on the chromosome were tested for uniform periplasmic localization of the fusion protein and foci formation after the addition of external transforming DNA (A), for DNA uptake (B) as described for Fig. 2, or for natural transformation (C). The transformation frequencies shown on the Y-axis are averaged from at least three independent replicates (± SD). <d.l., below detection limit (on average 3.4×10^−8^ ± SD of 1.1×10^−8^). Statistically significant differences were determined using Student's t-test on log-transformed values. **P<0.01.

To validate this model, we used site-directed mutagenesis to create ComEA variants with single or double amino acid substitutions. All *comEA-mCherry* alleles were inserted into the chromosome, thereby replacing the wild-type *comEA* copy. The ComEA-mCherry variants were tested for expression and periplasmic localization, foci formation upon provision of tDNA, for their ability to induce DNA translocation into a DNase resistant state (using the DNA uptake assay) and to restore natural transformation (Fig. 4 and Fig. S7). Consistent with the *in silico* predictions, K63 was of major importance. ComEA^K63A^ was severely impaired for natural transformation (∼250-fold reduction; Fig. 4C), resulting in DNA uptake levels below the limit of detection (Fig. 4B). The substitution of K63 with a negatively charged residue (ComEA^K63E^) or the concomitant exchange of K62 (ComEA^K62/63A^) completely abolished natural transformation (Fig. 4C). The ComEA-DNA model also explains why K63 has the major role in DNA binding: while K62 is engaged with a single backbone phosphate moiety, K63 is inserted into the DNA minor groove, chelating the backbone of both strands (Fig. 3B, inset). Moreover, a substitution of the nearby glycine residue at position 60 by alanine had no effect on DNA binding and transformation, whereas strains producing ComEA^G60V^ and ComEA^G60E^ were impaired in DNA uptake and were non-transformable (Fig. 4). We suggest that the combined effect of impairing the interactions of K62 and K63 with the dsDNA (as in the case of ComEA^G60E^) and perturbing the HhH1 GIG hairpin motif (Fig. 3A) has a major impact on the ability of ComEA to bind DNA.

To further investigate whether the lysine pair is indeed involved in DNA binding we heterologously expressed those variants as tat-ComEA-GFP fusions in *E. coli* (Fig. S8; as for wild-type ComEA in Fig. 2A). Using this approach we showed that the ComEA^K63E^ and ComEA^K62/63A^ variants behaved differently from WT ComEA in that they localized evenly within the cytoplasm. In addition, most of the *E. coli* cells did not show any compaction of the chromosome (and if so the variant did not co-localize with the compacted chromosome). The same phenotype was observable for variants that lacked either of the two HhH motifs (Fig. S8), suggesting that those variants had lost their ability to bind DNA. In contrast, a K63A variant showed an intermediate phenotype (Fig. S8) consistent with the ∼250-fold decreased transformation frequency observed for the ComEA^K63A^-mCherry variant in *V. cholerae* (Fig. 4).

Apart from this patch at HhH1, the only other amino acid important for the *in vivo* functionality of ComEA was the conserved arginine residue at position 71 (Fig. 3A). The DNA uptake ability of ComEA^R71A^ was slightly reduced, and less DNA-protein foci were observed for this variant (Fig. S7). However, the strain containing ComEA^R71A^ remained naturally transformable, a feature that was completely abolished for the ComEA^R71D^ variant. The latter mutant protein was also unable to bind DNA within the periplasmic space and did not foster the uptake of transforming DNA (Fig. S7). Based on our ComEA model structure, R71 is located in a position not particularly favorable for DNA binding (Fig. 3B); therefore, it is likely that R71 might be important for the structural stability of ComEA.

### *In vitro* binding of ComEA to DNA only occurs in the presence of the lysine pair K62/K63

To unambiguously show that the lysine residues are required for DNA binding we purified a tagged (*Strep*-tag II) version of ComEA, ComEA^K62/63A^, ComEA-mCherry, and ComEA^K62/63A^-mCherry (Fig. S9). The purified ComEA protein showed an unexpected UV-Vis spectrum, which was consistent with bound DNA (due to an absorption peak around 260 nm; Fig. S9A). Interestingly, if we compared purified ComEA-mCherry with the ComEA^K62/63A^-mCherry, we observed that the peak at 260 nm was absent in this variant, indicating that the protein was indeed no longer able to bind DNA.

**Figure 5:**
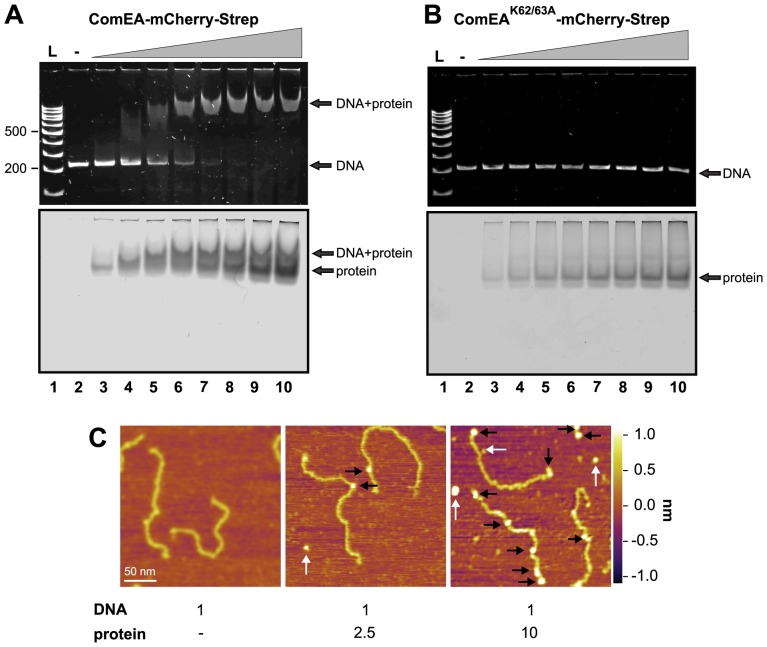
ComEA but not ComEA^K62/63A^ binds to DNA *in vitro*. EMSA using the 200*comEA* gene as a probe (panels A and B). A total of 0.4 pmol of DNA was incubated without or with increasing amounts of the ComEA-mCherry-Strep (A) and ComEA^K62/63A^-mCherry-Strep (B) protein (lanes 2 to 10: 0, 2, 4, 6, 8, 10, 12, 16, and 20 pmol of protein). Free DNA, free protein, and the DNA/protein complex are indicated by the arrows. L: DNA ladder (representative bp are indicated on the left). Panel C: AFM images of DNA, proteins, and DNA/protein complexes absorbed on mica. AFM images from left to right: bare DNA fragments (DNA to protein ratio 1∶0); DNA/protein complex at a molecular ratio of 1∶2.5; DNA/protein complex at a molecular ratio of 1∶10. The proteins bound to the DNA are marked with black arrows; unbound proteins are labeled by white arrows. The height or Z scale is shown on the right and is the same for all three panels displaying 270 nm×270 nm scan areas.

To remove any pre-bound DNA from the ComEA protein we included a DNase treatment step prior to the elution of the protein from the affinity column (see Material and Methods; Fig. S9C and D). All four proteins were tested for *in vitro* binding to DNA using an electrophoretic mobility shift assay (EMSA). Notably, ComEA-mCherry and ComEA bound to DNA in a concentration dependent manner as visualized by the retarded migration of the DNA probe (Fig. 5A and Fig. S10A) and the likewise changed migration of the protein (visualized by the fluorescence of mCherry; Fig. 5A). Notably, the K62/63A variants of ComEA did not change the migration behavior of the DNA probe (Fig. 5B and Fig. S10B), again confirming that the protein had lost the ability for DNA binding.

It should be noted that the shifted DNA signal was detectable at DNA to protein ratios as low as 1∶10 and the probe seemed completely shifted at a ratio of 1∶25–30 (Fig. 5A and Fig. S10A), which was significantly lower than what has been described for the *B. subtilis* ComEA homolog (98% of the DNA probe was shifted when 5.5×10^−11^ M of DNA was incubated with 1.6 µM of purified protein; [21]) or for the neisserial ComE ortholog [23]. A possible explanation for this difference could be that the ComEA/ComE proteins investigated in those earlier studies were pre-occupied by DNA as we observed for ComEA of *V. cholerae* in the absence of DNase treatment.

### *V. cholerae* ComEA does not show cooperativity for DNA binding

Provvedi and Dubnau suggested that the *in vitro* DNA binding behavior of the ComEA protein of *B. subtilis* was indicative of cooperative binding [21]. To test whether any cooperative binding was observable for ComEA of *V. cholerae* we used Atomic Force Microscopy (AFM). AFM allows investigating the extent of ComEA-mCherry binding to a DNA fragment and to also determine where on the DNA the protein is bound (e.g. fractional occupancies at any specific site, binding to the ends, or to nonspecific sites). To minimize overestimation of the binding affinity that can occur in the case when coverage of protein on the surface is too high, such that the protein coincidently lands on DNA, we kept the DNA-protein molecular ratio low by not exceeding a ratio of 1∶10 (DNA to protein). Prior to AFM imaging, we pre-incubated the ComEA-mCherry protein with a random PCR fragment (809 bp) at a molecular ratio of 1∶2.5 or 1∶10. As illustrated in Fig. 5 we observed a mixture of bare DNA molecules, free protein molecules, and protein/DNA complexes. To identify the ComEA-mCherry protein in topographic AFM images we used height and width criteria (height >2 nm, width from 10 to 20 nm). Using an approach reported by Yang *et al.* [40] we found that the probability of protein molecules located on DNA was 5 times higher than it would be for stochastically binding of the protein to the mica surface. Moreover, in the case of a DNA to protein ratio of 1∶10 we observed 2.5-fold higher affinity of the protein to the free ends of DNA than to random sites on the DNA strand. These AFM data indicate that, at least at the measured concentrations, no cooperative binding of the ComEA protein to DNA occurred and again contradicts the hypothesis that binding of ComEA might primarily protect the tDNA from degradation. Such protective effect has been demonstrated for the competence protein DprA of *Streptococcus pneumoniae* [41], which binds the single-stranded tDNA after its translocation into the cytoplasm. Indeed, Mortier-Barrière *et al.*, described in their study that DprA binding to DNA appeared to be cooperative since fully covered protein-DNA complexes were observed next to free ssDNA molecules at a protein to nucleotide ratio of 1∶20. We never observed such scenario for ComEA's binding to dsDNA using AFM (though we used a ∼4-fold lower protein to nucleotide ratio).

### Entry of DNA into the periplasm of naturally competent *V. cholerae* cells occurs at one distinct location

**Figure 6:**
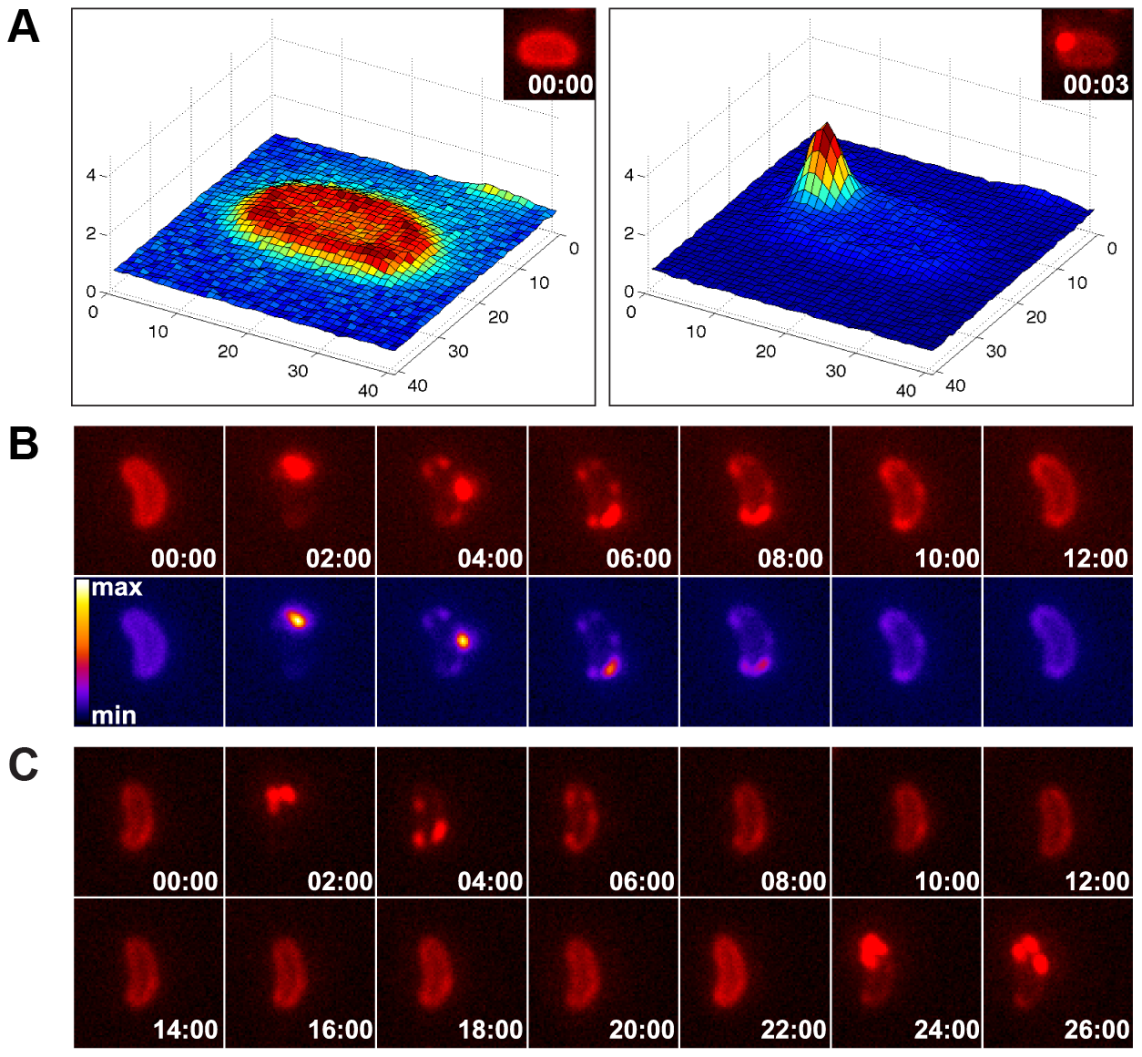
Time-lapse microscopy series of bacteria exposed to exogenous DNA. The images were captured in the red channel to visualize ComEA-mCherry at intervals of 3 sec (A) or 2 min (B). Matlab-computed maximal fluorescence intensity-plots are shown in A (corresponding fluorescent image in inset). Heat-maps showing the fluorescence intensities of the mCherry signal are depicted in the lower row of panel B. (C) Time-lapse microscopy series as in (B), but in a *comEC* minus background. The corresponding movies are available online (movies S2, S3, S4).

Interestingly, a passive DNA uptake mechanism has recently been proposed for single-stranded T-DNA translocation into plant cells involving the VirE2 protein of *Agrobacterium tumefaciens* [42]. We reasoned that if a similar mechanism is responsible for DNA uptake in competent *V. cholerae* cells, although dsDNA is involved and ComEA shows no similarity to VirE2, then the aggregation of ComEA should occur at one distinct DNA entry point (most likely next to the PilQ secretin). To test this hypothesis, we performed time-lapse microscopy experiments using ComEA-mCherry-expressing *V. cholerae* strains in the presence of external DNA (Fig. 6). We consistently observed the accumulation of ComEA as one large focus before smaller subclusters separated from the main ComEA focus and spread throughout the periplasm until the uniform localization of ComEA was restored (Fig. 6, movies S2, S3, S4).

### The function of ComEA might be conserved among naturally competent bacteria

**Figure 7:**
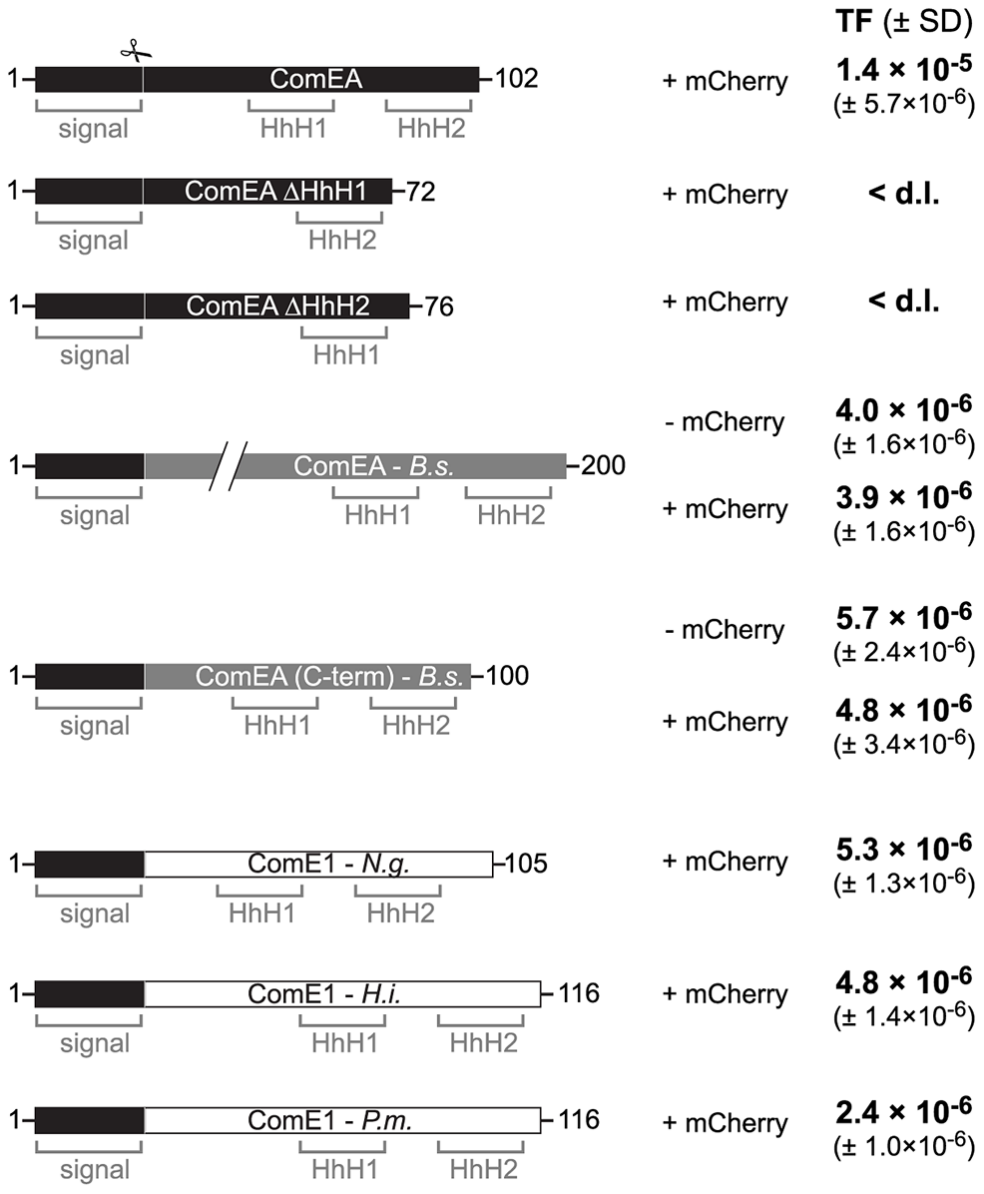
ComEA homologs from different naturally competent bacteria compensate for the absence of ComEA in *V. cholerae*. First row: Schematic representation of the ComEA protein of *V. cholerae* from amino acid 1 to the end. Domains such as the helix-hairpin-helix motifs (HhH) and the signal sequence (signal) are also depicted. The signal sequence cleavage site (scissor) was predicted through the SignalP 4.1 server [73] for ComEA*^V.c.^* and for its homologs. Designed constructs replacing wild-type ComEA on the chromosome of the respective *V. cholerae* strains are indicated below the WT ComEA scheme. First, the two ComEA mutants lacking either of both HhH motifs are indicated. In gray: the ComEA protein of *B. subtilis* (*B.s.*; not to scale compared to the other constructs and as indicated by the two diagonal lines). The transmembrane domain of ComEA*^B.s.^* was removed to avoid toxicity/insolubility problems [21]. ComEA(C-term) of *B. subtilis* refers to the C-terminal part of the protein, which still allowed DNA binding *in vitro* [21]. Both ComEA*^B.s.^* and ComEA(C-term)*^B.s.^* were tested without and with mCherry fused to the C-terminus (− mCherry/ + mCherry). In white: ComEA homologs (ComE1) of the Gram-negative bacteria *Neisseria gonorrhoeae* (*N.g.*), *Haemophilus influenzae* (*H.i.*), and *Pasteurella multocida* (*P.m.*). The original signal sequence of each of those proteins was removed. All constructs were expressed from the native *comEA* promoter and encoded the *V. cholerae* ComEA-specific signal sequence (residues 1–25) to allow proper translocation across the inner membrane. Natural transformation was tested for all strains and the transformation frequencies (TF) are indicated on the right. The average of at least three independent biological replicates is shown (± SD). <d.l., below detection limit.

Based on the data presented above we hypothesize that ComEA might play a direct role in the translocation of DNA across the outer membrane solely based on its ability to bind to DNA. If this were the case then ComEA homologs of other naturally competent bacteria should be able to replace ComEA of *V. cholerae*. And indeed, ComEA of *B. subtilis* was able to efficiently compensate for the absence of ComEA of *V. cholerae* (Fig. 7). Moreover, even the C-terminal (HhH)_2_ motif of ComEA of *B. subtilis* alone, which was shown to bind DNA *in vitro* [21], was sufficient to restore natural transformation of a *comEA* negative *V. cholerae* strain as were the ComEA homologs from *N. gonorrhoeae, Haemophilus influenzae*, and *Pasteurella multocida* (Fig. 7). It should be noted that Sinha *et al.* suggested that *H. influenzae* might contain an additional but so far unidentified paralog of *comE1* due to the modest effect observed for a *comE1* minus strain [43].

It is tempting to speculate that ComEA might fulfill a similar role in Gram-positive bacteria. Indeed, the localization of ComEA has been previously described for *B. subtilis* [16], [17], [19] but those studies were either based on immunofluorescence microscopy [16], which does not allow following protein localization over time, or were done in the absence of tDNA [17], [19]. Therefore, it was concluded by Kaufenstein *et al.* that ComEA localizes to many sites of the cell membrane and only occasionally co-localizes with the polar DNA uptake machinery, which was mainly achieved by changing the artifical inducer concentration [19]. However if the cell wall would be considered as a similar barrier in Gram-positive bacteria as the outer membrane is in Gram-negatives, creating a kind of periplasmic space between the cell wall and the (inner) membrane as suggested by Matias and Beveridge [44], then the binding of ComEA could also participate in the transport of DNA across the cell wall layer. However, in contrast to ComEA of Gram-negative bacteria, ComEA of Gram-positives is anchored to the membrane and therefore accumulation of ComEA can only occur in two dimensions, which might still be sufficient to prevent backward diffusion of the tDNA and contribute to DNA translocation across the cell wall. Notably, while this article was under revision Bergé *et al.* published a study on the nuclease EndA of naturally competent *Streptococcus pneumoniae* [45]. The authors demonstrated that EndA aggregates at midcell in this Gram-positive bacterium and that this recruitment is dependent on “the dsDNA receptor” ComEA [45]. Interestingly, ComEA also localized to the midcell and the authors speculated “a direct interaction of EndA and ComEA, an hypothesis which received indirect support” [45].

### A working model for ComEA-dependent DNA translocation across the outer membrane

**Figure 8:**
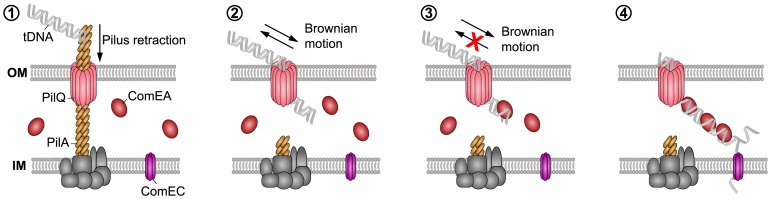
Working model of how DNA translocation across the outer membrane might occur in *V. cholerae* (based on the current study and [2]–[4], [13], [14]). The key components addressed in this study are indicated. It has been suggested that a (pseudo)pilus [1], [2], which is similar to type 2 secretion systems (T2SS) and type IV pili (Tfp), represents a core element of the DNA uptake machinery [11]. It is assumed that the Tfp crosses the outer membrane through a secretin pore formed by PilQ [2], [4], [74], [75]. This secretin could also provide the point of entry for incoming DNA. A single pilus retraction event might open the secretin pore so that short stretches of the tDNA can enter the periplasm by Brownian motion (or through partial binding to the pilus structure). ComEA would then bind to the tDNA via its HhH-associated lysine residues favoring translocation via a Brownian ratchet mechanism, which is modulated by the effective ComEA DNA-binding kinetics, binding spacing, and concentration. The ComEA-loaded tDNA might eventually interact with the inner membrane transporter ComEC [76], which would transport the DNA into the cytoplasm. Similar to Gram-positive bacteria, it is assumed that incoming DNA enters the cytoplasm of Gram-negative bacteria single-stranded.

Our findings suggest that the ability of ComEA proteins to bind to dsDNA emerging from the PilQ pore can potentially prevent the retrograde movement of the substrate, and ComEA binding might contribute to pull DNA into the periplasm (Fig. 8). It has been suggested that ratcheting produced through binding proteins can significantly accelerate translocation events [46], [47], as for the case of phage DNA injection into bacterial cells [15]. Based on our data, a similar mechanism can be envisioned for the ComEA-mediated transfer of DNA into the periplasm, with the rate of uptake depending on the specific binding kinetics and concentration of ComEA [15]. We hypothesize that ComEA-mediated DNA internalization might start occurring once short stretches of tDNA would enter the periplasm (most likely through the outer membrane secretin PilQ and potentially after a single Tfp retraction event). The ratio between the periplasmic ComEA protein and the incoming tDNA should be high at that stage thereby leading to an increased ComEA effective binding density, which, potentially together with the higher affinity of ComEA for DNA ends as observed by AFM (Fig. 5), would promote efficient DNA internalization. The absence of cooperative ComEA-DNA binding revealed by our AFM data (Fig. 5) is not an obstacle to a ComEA-mediated ratchet mechanism of internalization, as cooperativity would only contribute to increase the relative speed of the process [15], [46], [47]. The binding of proteins has undeniably been recognized as a driving force, both in the translocation of proteins as well as of DNA [48]. To this extent, Salman *et al.* investigated the translocation of double-stranded (ds) DNA through the nuclear pore complex using a combination of epifluorescence microscopy and single-molecule manipulation techniques [49]. They presented evidence that the DNA uptake process in their reconstituted system was based on a passive ratchet, directed by the retention of the already translocated segment of the DNA [49]. We suggest that ComEA might play a similar role in the DNA uptake process in naturally competent *V. cholerae* cells.

In summary, we used a cell biological approach to better understand DNA uptake in naturally competent *V. cholerae* cells. We visualized the competence protein ComEA and observed the *in vivo* binding of this protein to dsDNA in real time. Structural modeling and AFM experiments suggested that the binding of ComEA to DNA is primarily responsible for DNA translocation across the outer membrane. Consistent with this suggestion, ComEA variants unable to bind to DNA *in vivo* were also defective in promoting DNA uptake and natural transformation. We hypothesize that ComEA encounters incoming DNA immediately after short stretches of DNA have crossed the outer membrane (through the PilQ secretin or in exceptional cases also in a Tfp-independent manner [11]) and that ComEA subsequently promotes DNA translocation across the outer membrane without the need for any external energy source (Fig. 8). ComEA might therefore be more than a DNA receptor protein, but rather a crucial player for mediating DNA uptake in *V. cholerae* and potentially also other naturally competent bacteria.

## Materials and Methods

### Bacterial strains, plasmids, and culture conditions

*Vibrio cholerae* strains and plasmids used in this study are listed in Table S1. *Escherichia coli* strain DH5α [50] was used as host for cloning purposes and for heterologous expression of ComEA and its variants for protein purification. Genomic DNA (gDNA) extracted from *E. coli* BL21 (DE3) [51] was utilized to test DNA uptake by PCR as described [24]. *E. coli* S17-1λpir [52] served as donor strain for bacterial mating with *V. cholerae*.

All *V. cholerae* and *E. coli* strains were grown aerobically in Luria-Bertani (LB) medium at 30°C and 37°C, respectively. Solid LB plates contained 1.5% agar. For *tfoX* expression and induction of other constructs under control of the P_BAD_ promoter the LB medium was supplemented with 0.02% L-arabinose (L-ara). For expression of *tat-gfp*, *tat-comEA-gfp*, and its derivatives in *E. coli* DH5α (Fig. 2A and Fig. S8) L-ara concentrations were lowered to 0.002%. Thiosulfate Citrate Bile Salts Sucrose (TCBS) agar plates were prepared following the manufacturer's instructions (Fluka) and used to counterselect *E. coli* after bacterial mating. For sucrose-based counterselection, NaCl-free LB medium containing 6% sucrose was used. LB medium and LB agar plates were supplemented with antibiotics when required. Final concentrations of antibiotics were 50 µg/ml, 75 µg/ml and 100 µg/ml for gentamicin, kanamycin, and ampicillin, respectively. The ampicillin concentration was lowered to 50 µg/ml for *V. cholerae* strains induced for competence.

### Recombinant DNA techniques

Standard molecular biology-based methods were used for DNA manipulations. Restriction enzymes and DNA modifying enzymes were obtained from New England Biolabs, Taq DNA polymerase (GoTaq) was obtained from Promega and used for colony PCR, and Pwo DNA Polymerase (Roche) was used for high-fidelity PCR amplifications. Modified DNA sequences were verified using Sanger sequencing (Microsynth, CH).

### Plasmid construction

All plasmid constructs were based on pBAD/Myc-HisA (Invitrogen), which contains the *ara*BAD (P_BAD_) promoter followed by a multiple cloning site (MCS) for dose-dependent protein expression. A derivative of pBAD/Myc-HisA, pBAD(kan), was created through substitution of the ampicillin resistance cassette (*bla*) with a kanamycin resistance cassette (*aph*). The genes and translational fusion constructs were PCR amplified and cloned into the MCS of pBAD/Myc-HisA or pBAD(kan). For the amplification of *V. cholerae* genes, the gDNA of strain A1552 [53] served as a template. The accuracy of the plasmids was verified through sequencing.

### Strain constructions

Genes were deleted from the parental strain A1552, using either a gene disruption method based on the counter-selectable plasmid pGP704-Sac28 [54], or natural transformation and FLP recombination, as recently described (TransFLP method [55]–[57]).

Strains containing *comEA-mCherry* or site-directed variants thereof were constructed using the TransFLP method [55]–[57]. For the construction of ComEA site-directed variants, a silent ‘watermark’ restriction site was inserted close to or including the changed nucleotide sequence. This watermark simplified screening purposes after homologous recombination.

The *comEA^B.s.^* gene (or parts thereof) was amplified from gDNA derived from *B. subtilis* strain 168. The DNA fragment containing *comE1* from *Neisseria gonorrhoeae (N.g.*; *Neisseria gonorrhoeae* strain FA 1090, NCBI Reference Sequence: NC_002946.2; locus YP_208252), *Haemophilus influenzae* (*H.i.*; *Haemophilus influenzae* strain R2846, NCBI Reference Sequence: NC_017452.1; locus YP_005829750), and *Pasteurella multocida* (*P.m.*; *Pasteurella multocida subsp. multocida* str. Pm70, NCBI Reference Sequence: NC_002663.1; locus NP_246604, hypothetical protein PM1665) was synthesized using the GeneArt® Strings^™^ technology (Life technologies/Invitrogen) and served as PCR template for the TransFLP strain construction method [55]–[57]. The beta-lactamase gene (*bla*) was amplified from plasmid pBR-flp [55]–[57]. All strains were verified through colony PCR (in part followed by restriction enzyme digestion according to inserted watermarks) and confirmed through PCR amplification and sequencing.

### Wide-field fluorescence microscope settings and image analysis

Microscopy images were obtained using a Zeiss Axio Imager M2 epifluorescence microscope. Details about the instrumentation and configurations are provided elsewhere [25]. All bacterial samples were mounted on 2% agarose/PBS pads. Image processing and annotation was done using ImageJ and Adobe Illustrator.

### Microscopy of strains expressing fluorescent fusion proteins

Strains carrying fluorescent fusion constructs were grown aerobically for ∼5 h in LB supplemented with the respective antibiotics and 0.02% L-arabinose (0.002% L-ara for *E. coli* experiments; Fig. 2 and Fig. S8). The strains carrying chromosomally encoded fluorescent fusion proteins were grown aerobically and at 30°C in LB supplemented with 0.02% L-ara for ∼7 h (OD_600_ 2.5; [11]). The samples were washed once in PBS and immediately imaged.

The staining of chromosomal DNA was performed through the addition of 4′,6-diamidino-2-phenylindole (DAPI; final concentration 5 µg/ml) to the bacterial cultures for at least 5 min.

To characterize the ComEA-mCherry localization dynamics during DNA uptake, *comEA-mCherry*-expressing strains were grown as described above. A total of 50 µl of washed culture was mixed with 1 µg of either gDNA derived from *V. cholerae* strain A1552-lacZ-Kan [58], commercially available phage lambda DNA (Roche) or a 10.3 kb fragment amplified through PCR. After 5 min of incubation with the DNA the bacteria were mounted on agarose pads and imaged. To visualize the DNA during the relocalization of ComEA-mCherry, phage lambda DNA (Roche) was pre-stained with 10 µM YoYo-1 (Molecular Probes/Invitrogen) at 4°C corresponding to a base pair to dye ratio of 15∶1. The bacterial culture was mixed with the pre-stained DNA and incubated for 20 min. The cells were washed in PBS, mounted on agarose pads and imaged.

For time-lapse microscopy, the samples were prepared as described above, but immediately imaged after the addition of DNA. The images were taken every 3 or 120 sec as indicated in the figure and movie legends. For time-lapse imaging, the agarose pads were sealed using a mixture of Vaseline, lanolin and paraffin (VALAP).

### Fluorescence loss in photobleaching

Fluorescence loss in photobleaching (FLIP) experiments were performed on a Zeiss LSM710 microscope equipped with a 561 nm solid-state laser (20 mW). A Plan-Apochromat 63×/1.40 Oil objective was used. The microscope was controlled with the Zen 2009 software suite (Zeiss). Time intervals ranged from 104 to 120 ms/frame for live cells to max. 160 ms/frame for fixed cells. The maximum (100%) laser power was used for bleaching.

*V. cholerae* strains ΔcomEA-Tn*tfoX* harboring pBAD(kan)-*comEA*-mCherry or pBAD(kan)-ss[ComEA]-mCherry were grown aerobically for 5 h in LB supplemented with 0.02% arabinose and 75 µg/ml of kanamycin. After the cells were mounted, the slides were sealed and the bacteria were immediately imaged (live samples; Fig. 1 and Fig. S2A). Alternatively, the cells were fixed for 30 min (4% paraformaldehyde/150 mM phosphate buffer) before imaging (fixed samples; Fig. S2B).

For FLIP data acquisition a circular bleaching region of ∼440 nm width was defined at one cell pole (region-of-interest (ROI) 1; labeled as 1 in Fig. 1). A circular ROI of the same size was defined at the opposite cell pole of the same bacterium (labeled as 2 in Fig. 1) and in an adjacent cell (labeled as 3 in Fig. 1). The average fluorescence intensity of all regions was recorded. Bleaching of ROI 1 was initiated after a lag of 20 frames and repeated after each frame. The acquired data were exported and processed in ‘R’ [59]. The recorded fluorescence intensities were normalized to the average fluorescence intensity of the first 10 frames. Moving averages were calculated using the SMA(x, n = 5) function from the ‘TTR’ package [59].

### Natural transformation assays

Transformation assays were performed as previously described [25] with gDNA of strain A1552-lacZ-Kan [58] as transforming material. Transformation frequencies were calculated as the number of transformants divided by the total number of colony forming units (CFU). Differences in transformation frequencies were considered significant for *P*-values below 0.05 (*) or 0.01 (**) as determined by Student's t-test on log-transformed data.

### Detection of DNA uptake by PCR

DNA uptake was verified using a whole-cell duplex PCR assay as described [24] with slight modifications. Briefly, competence-induced bacteria were grown aerobically until an OD_600_ of 1.0–1.5 before genomic DNA (gDNA) (2 µg/ml) of *E. coli* strain BL21 (DE3) was added for 2 h. For the uptake of YoYo-1-labeled DNA gDNA of *E. coli* strain BL21 (DE3) was pre-labeled as described for the microscopy experiments and YoYo-1 was maintained in the solution throughout the 2 h incubation period. Next, cells were harvested and treated with DNase I (Roche) for 15 min at 37°C. Excess nuclease was removed by washing and cells were resuspended in 100 µl PBS. ∼3×10^6^ bacteria were used as template in a whole-cell duplex PCR. Primer pairs were specific for the donor DNA derived from *E. coli* BL21 (DE3) and for gDNA of the *V. cholerae* acceptor strain (at a 10-fold lower concentration). The latter reaction served as control for the total number of acceptor bacteria [24].

### 3D modeling of ComEA, its interaction with dsDNA, and molecular simulations

A 3D model structure was produced for ComEA (truncating the first 37 residues including the 25 residue-containing signal peptide) using comparative modeling (MODELLER package [60]) on the *Thermus thermophilus* HB8 (PDB ID: 2DUY) template (with 43% sequence identity) (Fig. 3). The ComEA-DNA complex was modeled, to identify structurally similar DNA-binding proteins using the DALI server [39]. The DNA polymerase, PolC, from *Geobacillus kaustophilus* (PDB ID: 3F2D) [61] was selected as the best match, with 24% sequence identity and a root mean square deviation (RMSD) of 2.4 Å compared with the modeled ComEA of *V. cholerae*. The PolC X-ray structure complexed with DNA was used to identify potential DNA poses on the *V. cholerae* ComEA model using the Chimera MatchMaker tool [62]. This assessment led to the production of a DNA-ComEA model (Fig. 3B, movie S1), which was further refined and equilibrated using the minimization and molecular simulations detailed below. The estimated binding energy for the ComEA-DNA association is in the order of 29±8 kcal/mol, based on MM/PBSA calculation on the MD trajectory.

Molecular dynamics simulation was used to relax and study the dynamics and energetics of ComEA and the ComEA-DNA complex for 55 and 50 ns, respectively. The MD simulations were run using the NAMD simulation package [63] with Amber force field (with Barcelona modification for nucleic acids [64] and the TIP3P water model [65]. The systems were first energy minimized using constrained C-alpha atoms, followed by analysis without any constraint for 2000 steps. To equilibrate the system, the temperature was gradually increased up to 300 K in the NVT ensemble and maintained at 300 K for 100 ps with a 1 fs time step. Finally, an NPT simulation was run at 300 K for 500 ps with a 2 fs time step to complete the equilibration procedure. The equilibrated structure was used as starting point for production simulations. All production MD simulations were run at 1 bar with a time step of 2 fs, using SHAKE algorithm [66] on all bonds and PME [67] for treating electrostatic interactions. To control the temperature and the pressure, Langevin dynamics and the Nose-Hoover Langevin piston, respectively, were used [68], [69]. The trajectories were saved every 500 steps in the production simulations. To characterize the binding affinity of different systems, the free binding energies were calculated using the MMPBSA.py package [70]. 100 frames were sampled from the trajectories for analysis using MMPBSA.py. The entropy portion of the free energy was not considered in the calculation. In addition, the PME module in VMD was used to estimate the electrostatics potential of the modeled ComEA monomers (Fig. 3C).

### Purification of recombinant ComEA and its variants

ComEA, ComEA^K62/63A^, ComEA-mCherry, and ComEA^K62/63A^-mCherry (all containing the eight amino acid *Strep*-tag II sequence at the C-terminus) were purified as previously described [71] with minor modification. Briefly, *E. coli* cells containing the respective plasmids (Table S1) were grown aerobically at 37°C until an OD_600_ of 1.0. At that time expression was induced by the addition of 0.2% arabinose to the culture medium and the cells were further incubated for 2 hours before their harvest at 4°C and storage of the cell pellet at −80°C. The cells were lysed by sonication (Vibra-cell; 10 min. in total with 30 sec on and 30 sec off intervals and an amplitude of 80%) and the lysate was further processed as described [71]. Notably, after realization that the protein was pre-occupied by DNA (see results section), we included a on-column DNase treatment step (10 µg/ml of DNase I (Roche) in 100 mM Tris/HCl pH 8.0 buffer containing 20 mM MgCl_2_ and 0.2 mM CaCl_2_; 30 min. at 30°C) after the soluble protein fraction was loaded onto the streptactin resin and washed with 5 column volumes of washing buffer. The DNase I treatment step was followed by extensive washing of the column (10 to 30 volumes) before the respective protein was eluted as described [71]. The eluted proteins were concentrated using Amicon Ultra spin columns (with a MWCO of 3 kDa or 10 kDa; Millipore). For the AFM experiment, the protein was dialyzed against AFM buffer (5 mM Tris/HCl pH = 8.0 and 10 mM MgCl_2_). The protein concentration was determined according to Bradford [72].

### Electrophoretic Mobility Shift Assays (EMSAs)

Electrophoretic Mobility Shift Assays were basically performed as previously explained [71]. However, as preliminary experiments indicated that neither the absence of DTT nor the storage of the protein in the absence of glycerol and at 4°C did change the results of the experiments, the protocol was changed accordingly. The 200 bp DNA fragment was PCR-amplified using gDNA of strain A1552 as template and represented the upstream region of the *comEA* gene. Other DNA fragments (e.g. the *aphA* promoter region as previously tested [71]) were similarly shifted (data not shown). The protein/DNA mixture was incubated for 5 min at room temperature before electrophoretic separation on an 8% polyacrylamide gel. DNA was visualized by ethidium bromide staining [71] whereas the fusion proteins (ComEA-mCherry and ComEA^K62/63A^-mCherry were detected using a Typhoon scanner (GE Healthcare; excitation at 532 nm (green) and emission detected with a 610 BP30 (red) filter).

### Atomic Force Microscopy (AFM)

To prepare the protein/DNA complex we mixed 0.85 ng/µl of a PCR-amplified DNA fragment (809 bp) with the protein in the molecular ratios of 1∶2.5 and 1∶10 (DNA∶protein) in buffer containing 5 mM Tris/HCl pH 8.0 and 10 mM MgCl_2_. After incubation for 10 min at 37°C, 15 µl of the mixture was deposited on freshly cleaved mica and rinsed thoroughly with ddH_2_O for two minutes. Preparation of the sample with bare DNA was done under the same conditions but in the absence of the protein. The AFM images were acquired in air and in tapping mode using an Asylum Research Cypher microscope. We used Olympus silicon cantilevers (Olympus OMCL-AC240TS-R3) with a spring constant of 1.7 N/m and a resonant frequency of 70 kHz. The typical scan rate was 2.0 Hz.

## Supporting Information

Figure S1 Periplasmic localization of ComEA. *V. cholerae* wild-type strain (A1552-Tn*tfoX*) and strain ComEA-bla-Tn*tfoX* (encoding a translational fusion between ComEA and beta-lactamase) were grown for 3 h at 30°C in LB medium in the absence or presence of the competence inducer L-arabinose (0.02 or 0.2% as shown on the left). Ampicillin (50 µg/ml) was added to the indicated cultures (+AMP) and growth of all cultures was resumed for 3 h. The protective effect of the ComEA-bla fusion protein located in the periplasm was checked by phase contrast imaging (boxed region).
(PDF)

Figure S2 Fluorescence loss in photobleaching (FLIP) experiment of ss[ComEA]-mCherry and of ComEA-mCherry in fixed cells. (A) Live *V. cholerae* cells expressing *ss*[ComEA]-*mcherry* (mCherry preceded solely by the signal sequence of ComEA; residues 1 to 25) were tested for mCherry mobility within the periplasmic space using FLIP. (B) The same bacterial strain as in Fig. 1B was tested, but the cells were fixed before microscopy. The settings for (A) and (B) were as described for Fig. 1B. Scale bars, 2 µm.
(PDF)

Figure S3 Localization of chromosomally encoded ComEA. The *comEA* gene of *V. cholerae* was replaced with the *comEA-mCherry* or the *ss*[ComEA]-*mCherry* allele using bacterial genetics (TransFLP [55]–[57]). The DNA was stained with DAPI. The fusion proteins were localized as in Fig. 1A. Images from left to right: mCherry channel (red), DAPI-stained chromosomal DNA (blue), overlaid fluorescent channels (merge), and phase contrast channel (Ph). Scale bar, 2 µm.
(PDF)

Figure S4 Expression and localization of ComEA-mCherry under chitin-inducing competence conditions. The *V. cholerae* strain harboring the *comEA-mCherry* translational fusion on the chromosome was grown on chitin surfaces for ∼24 h as described [25]. The bacteria were mounted for microscopy in the absence (left) or presence (right) of external gDNA. ComEA-mCherry was visualized in the red channel. The edge of the chitin bead is indicated with the dotted line. Scale bar, 2 µm.
(PDF)

Figure S5 ComEA is required for foci formation of YoYo-1-labeled DNA. (A) Visualization of YoYo-1-stained transforming DNA (green channel) in wild-type (WT), or in a *pilA* or *comEA* negative strain. The outline of the cells is shown in the phase contrast image (Ph). Scale bar, 2 µm. (B) DNA uptake assay using the indicated strains and YoYo-1-labeled tDNA as donor DNA. Details as in Fig. 2. L, ladder.
(PDF)

Figure S6 The absence of the nucleases Dns and Xds does not rescue the ΔcomEA phenotype. (A) Natural transformation of the indicated strains was scored using a chitin-independent transformation assay [25]. The transformation frequencies shown on the Y-axis are averaged from at least three independent replicates (± SD). <d.l., below detection limit. Statistically significant differences are indicated (**P<0.01); n.s. not statistically different. (B) DNA uptake assay as described for Fig. 2. The genotypes of the tested strains are indicated above the figure. L, ladder.
(PDF)

Figure S7 Localization and functionality of ComEA-mCherry variants. Additional ComEA-mCherry variants were tested for uniform periplasmic localization and the ability to aggregate after the addition of transforming DNA. (A) Table as in Fig. 4. (B) Representative images (for panel A and Fig. 4) showing uniform periplasmic localization, foci formation upon DNA binding, and DNA-independent aggregation (only observed for ComEA^N43I/N45A^). (C) DNA uptake assay of selected variants as described for Fig. 2. (D) Natural transformation assay as described for Fig. 4. The average of at least three independent biological replicates is shown (± SD). <d.l., below detection limit.
(PDF)

Figure S8 Localization of tat-ComEA-GFP variants. Variants tested: tat-ComEA-GFP (WT; A), tat-ComEA^K63A^-GFP (B), tat-ComEA^K63E^-GFP (C), tat-ComEA^K62/63A^-GFP (D), tat-ComEAΔHhH1-GFP (E), and tat-ComEAΔHhH2-GFP (F). Details are as in Fig. 2A. Scale bar, 2 µm.
(PDF)

Figure S9 Purification of ComEA, ComEA^K62/63A^, ComEA-mCherry and ComEA^K62/63A^-mCherry. ComEA and its variants (all containing a C-terminal *Strep*-tagII sequence) were purified by affinity chromatography. UV-Vis spectra of purified ComEA-Strep (A) or ComEA-mCherry-Strep (B, red line) and ComEA^K62/63A^-mCherry-Strep (B, back line) were recorded. Panel C and D: Purification of the ComEA-mCherry-Strep (C) and ComEA^K62/63A^-mCherry-Strep (D) protein followed by 11% SDS PAGE of the pooled fractions at each step. Lane 1, molecular mass (kDa) standard; lanes 2 to 10: cell lysate of the respective *E. coli* strains before and after induction, S17 extract, aliquots of the last two washing steps after on-column DNase treatment, and elution fractions 1 to 4. The gels were stained with Coomassie. The respective UV-Vis spectra are indicated below the gel images.
(PDF)

Figure S10 ComEA behaves similar as ComEA-mCherry in EMSA. EMSA were performed using purified ComEA-Strep (A) and ComEA^K62/63A^-Strep (B). Details as described in Fig. 5.
(PDF)

Movie S1 3D model of ComEA based on comparative modeling using the ComEA-related protein of *T. thermophilus* HB8 (PDB ID: 2DUY) as template and its predicted DNA binding site. Residues K62, K63 and R71 are highlighted (as in Fig. 3).
(MPG)

Movie S2 Time-lapse microscopy series of *V. cholerae* exposed to exogenous DNA. Images were taken in the red channel to visualize ComEA-mCherry at intervals of 3 sec (as in Fig. 6A).
(MOV)

Movie S3 Time-lapse microscopy series in the presence of exogenous DNA. Images were taken in the red channel to visualize ComEA-mCherry at intervals of 2 min (as in Fig. 6B).
(MOV)

Movie S4 Time-lapse microscopy series of a *V. cholerae* strain lacking the inner membrane channel ComEC (ΔcomEC). Images were taken in the red channel to visualize ComEA-mCherry at intervals of 2 min (as Fig. 6C). Please note that two DNA uptake events occur within the total duration of 36 min.
(MOV)

Table S1 Bacterial strains and plasmids.
(DOCX)
