# Supplementary figures and images for: ComEA Is Essential for the Transfer of External DNA into the Periplasm in Naturally Transformable Vibrio cholerae Cells
Source: PLoS Genet. 2014 Jan 2;10(1):e1004066. doi: 10.1371/journal.pgen.1004066 (PMC3879209; doi:10.1371/journal.pgen.1004066)

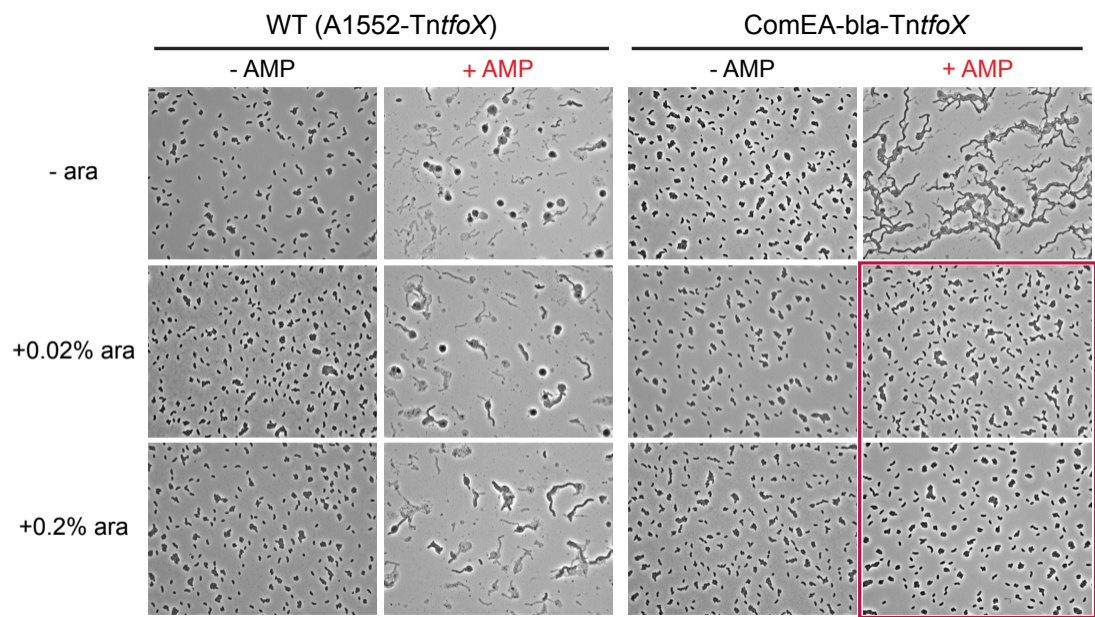

**Fig. S1**

Supplement: Figure S1 — Periplasmic localization of ComEA. V. cholerae wild-type strain (A1552-TntfoX) and strain ComEA-bla-TntfoX (encoding a translational fusion between ComEA and beta-lactamase) were grown for 3 h at 30°C in LB medium in the absence or presence of the competence inducer L-arabinose (0.02 or 0.2% as shown on the left). Ampicillin (50 µg/ml) was added to the indicated cultures (+AMP) and growth of all cultures was resumed for 3 h. The protective effect of the ComEA-bla fusion protein located in the periplasm was checked by phase contrast imaging (boxed region). (PDF) [file pgen.1004066.s001.pdf]

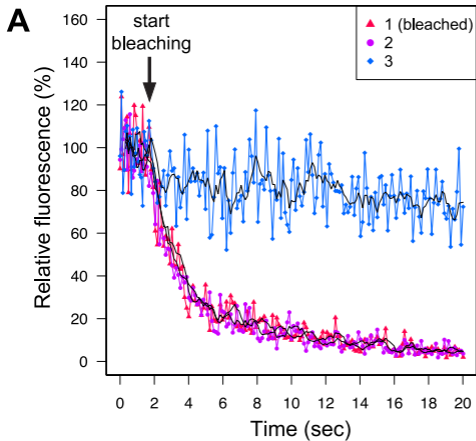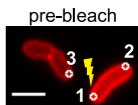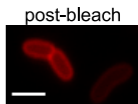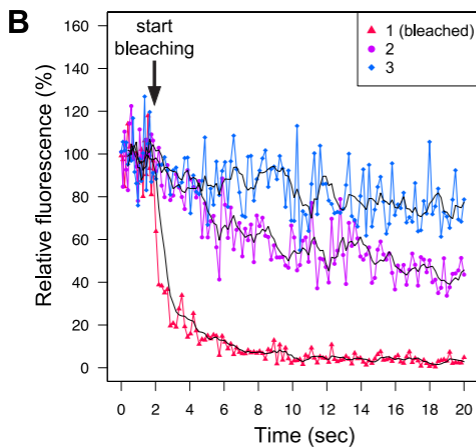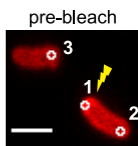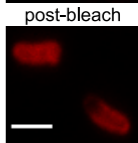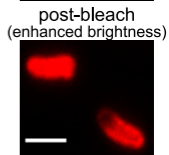

**Fig. S2**

Supplement: Figure S2 — Fluorescence loss in photobleaching (FLIP) experiment of ss[ComEA]-mCherry and of ComEA-mCherry in fixed cells. (A) Live V. cholerae cells expressing ss[ComEA]-mcherry (mCherry preceded solely by the signal sequence of ComEA; residues 1 to 25) were tested for mCherry mobility within the periplasmic space using FLIP. (B) The same bacterial strain as in Fig. 1B was tested, but the cells were fixed before microscopy. The settings for (A) and (B) were as described for Fig. 1B. Scale bars, 2 µm. (PDF) [file pgen.1004066.s002.pdf]

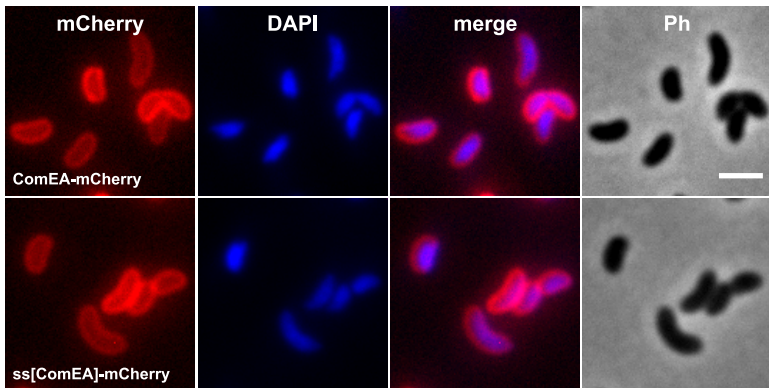

**Fig. S3**

Supplement: Figure S3 — Localization of chromosomally encoded ComEA. The comEA gene of V. cholerae was replaced with the comEA-mCherry or the ss[ComEA]-mCherry allele using bacterial genetics (TransFLP [55]–[57]). The DNA was stained with DAPI. The fusion proteins were localized as in Fig. 1A. Images from left to right: mCherry channel (red), DAPI-stained chromosomal DNA (blue), overlaid fluorescent channels (merge), and phase contrast channel (Ph). Scale bar, 2 µm. (PDF) [file pgen.1004066.s003.pdf]

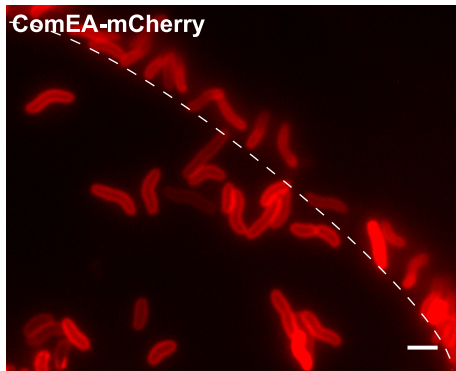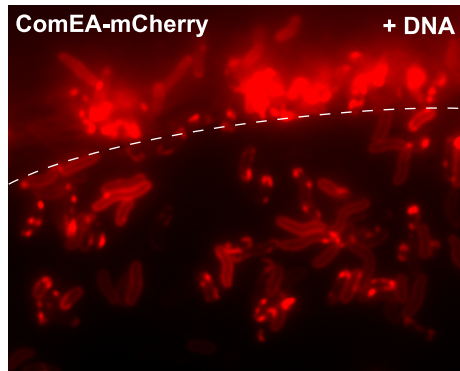

**Fig. S4**

Supplement: Figure S4 — Expression and localization of ComEA-mCherry under chitin-inducing competence conditions. The V. cholerae strain harboring the comEA-mCherry translational fusion on the chromosome was grown on chitin surfaces for ∼24 h as described [25]. The bacteria were mounted for microscopy in the absence (left) or presence (right) of external gDNA. ComEA-mCherry was visualized in the red channel. The edge of the chitin bead is indicated with the dotted line. Scale bar, 2 µm. (PDF) [file pgen.1004066.s004.pdf]

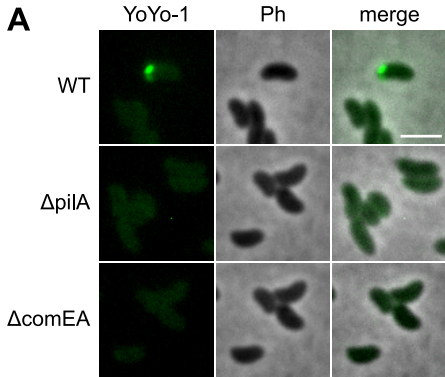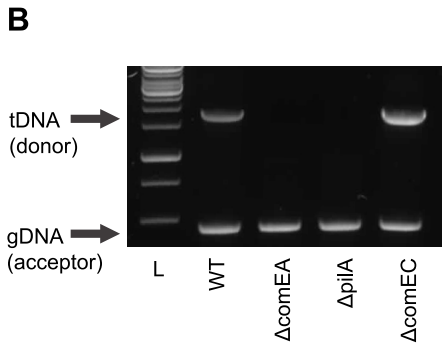

**Fig. S5**

Supplement: Figure S5 — ComEA is required for foci formation of YoYo-1-labeled DNA. (A) Visualization of YoYo-1-stained transforming DNA (green channel) in wild-type (WT), or in a pilA or comEA negative strain. The outline of the cells is shown in the phase contrast image (Ph). Scale bar, 2 µm. (B) DNA uptake assay using the indicated strains and YoYo-1-labeled tDNA as donor DNA. Details as in Fig. 2. L, ladder. (PDF) [file pgen.1004066.s005.pdf]

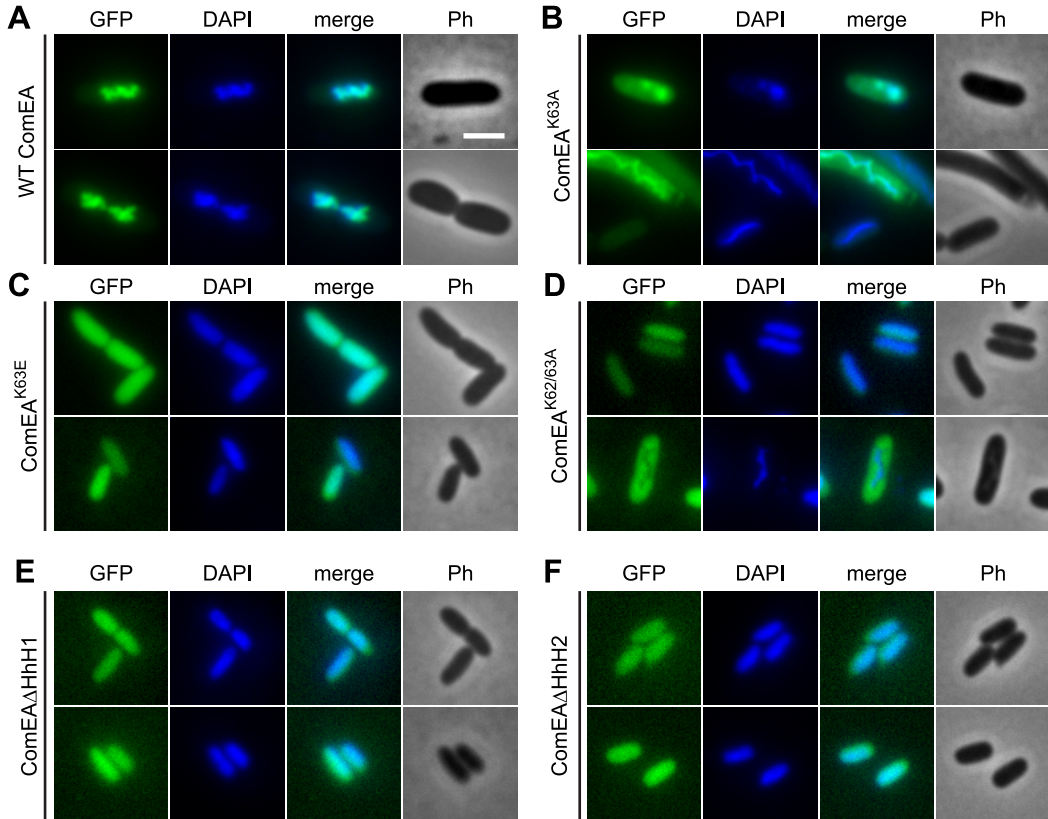

**Fig. S8**

Supplement: Figure S8 — Localization of tat-ComEA-GFP variants. Variants tested: tat-ComEA-GFP (WT; A), tat-ComEAK63A-GFP (B), tat-ComEAK63E-GFP (C), tat-ComEAK62/63A-GFP (D), tat-ComEAΔHhH1-GFP (E), and tat-ComEAΔHhH2-GFP (F). Details are as in Fig. 2A. Scale bar, 2 µm. (PDF) [file pgen.1004066.s008.pdf]

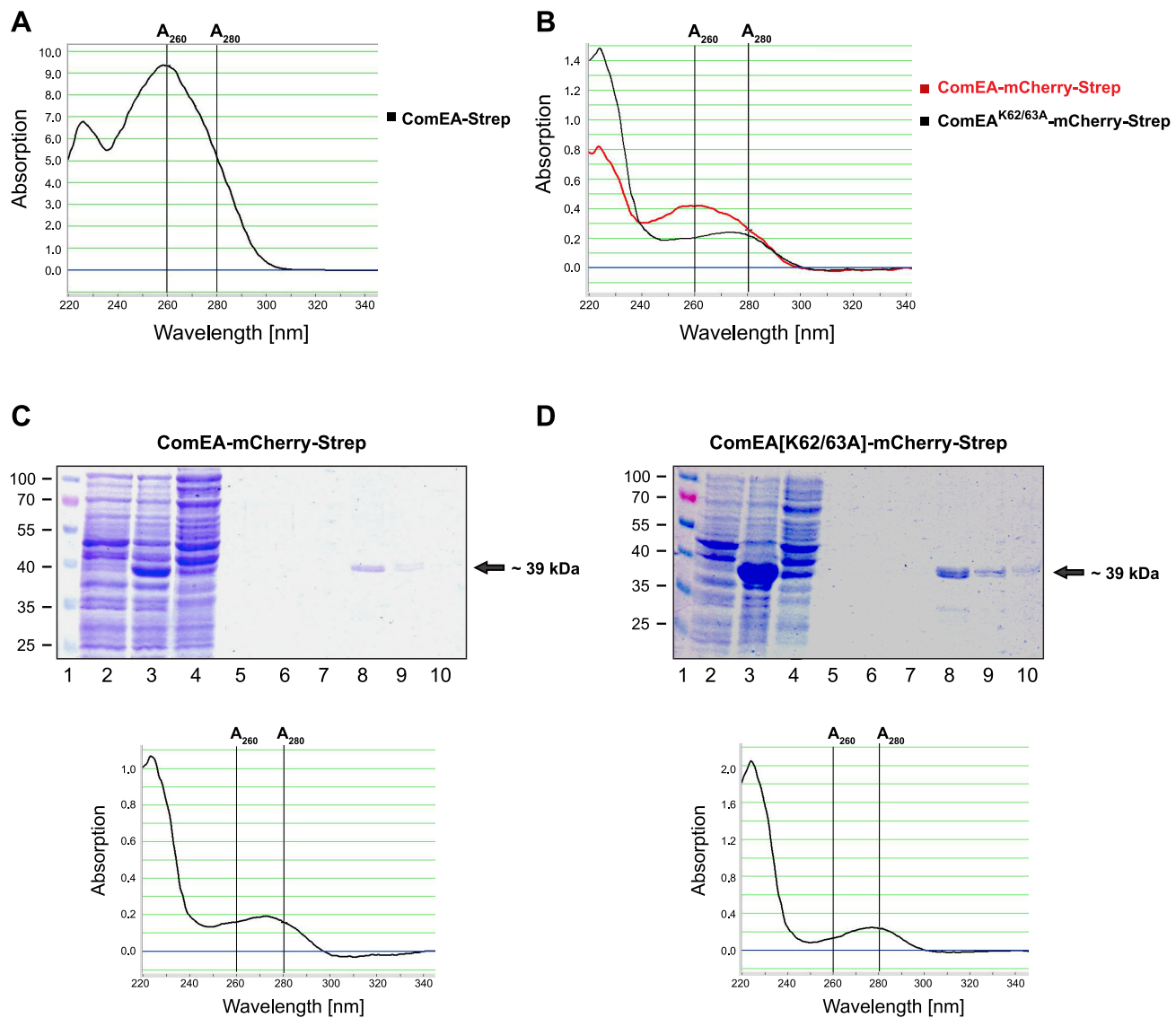

**Fig. S9**

Supplement: Figure S9 — Purification of ComEA, ComEAK62/63A, ComEA-mCherry and ComEAK62/63A-mCherry. ComEA and its variants (all containing a C-terminal Strep-tagII sequence) were purified by affinity chromatography. UV-Vis spectra of purified ComEA-Strep (A) or ComEA-mCherry-Strep (B, red line) and ComEAK62/63A-mCherry-Strep (B, back line) were recorded. Panel C and D: Purification of the ComEA-mCherry-Strep (C) and ComEAK62/63A-mCherry-Strep (D) protein followed by 11% SDS PAGE of the pooled fractions at each step. Lane 1, molecular mass (kDa) standard; lanes 2 to 10: cell lysate of the respective E. coli strains before and after induction, S17 extract, aliquots of the last two washing steps after on-column DNase treatment, and elution fractions 1 to 4. The gels were stained with Coomassie. The respective UV-Vis spectra are indicated below the gel images. (PDF) [file pgen.1004066.s009.pdf]

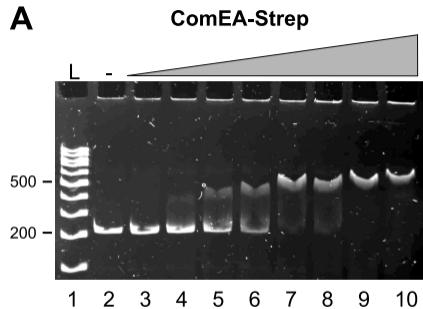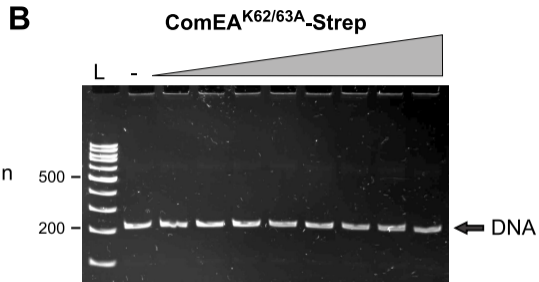

**Fig. S10**

Supplement: Figure S10 — ComEA behaves similar as ComEA-mCherry in EMSA. EMSA were performed using purified ComEA-Strep (A) and ComEAK62/63A-Strep (B). Details as described in Fig. 5. (PDF) [file pgen.1004066.s010.pdf]
