# Supplementary material for: ComEA Is Essential for the Transfer of External DNA into the Periplasm in Naturally Transformable Vibrio cholerae Cells
Source: PLoS Genet. 2014 Jan 2;10(1):e1004066. doi: 10.1371/journal.pgen.1004066 (PMC3879209; doi:10.1371/journal.pgen.1004066)
Supplement: Figure S7 — Localization and functionality of ComEA-mCherry variants. Additional ComEA-mCherry variants were tested for uniform periplasmic localization and the ability to aggregate after the addition of transforming DNA. (A) Table as in Fig. 4. (B) Representative images (for panel A and Fig. 4) showing uniform periplasmic localization, foci formation upon DNA binding, and DNA-independent aggregation (only observed for ComEAN43I/N45A). (C) DNA uptake assay of selected variants as described for Fig. 2. (D) Natural transformation assay as described for Fig. 4. The average of at least three independent biological replicates is shown (± SD). <d.l., below detection limit. (PDF) [file pgen.1004066.s007.pdf]

A

↓ feature strain* →	ComEA (WT)	ss[ComEA]	ComEA (N45A)	ComEA (N43I/N45A)	ComEA (S48A)	ComEA (S48T)	ComEA (E50A)	ComEA (E50R)	ComEA (E51A)
periplasmic localization	+	+	+	+	+	+	+	+	+
uniformly distributed	+	+	+	- #	+	+	+	+	+
foci formation + DNA	+	-	+	- #	+	+	+	+	+

↓ feature strain* →	ComEA (E51K)	ComEA (K57A)	ComEA (D69A)	ComEA (D69K)	ComEA (R71A)	ComEA (R71A/E72A)	ComEA (R71D)	ComEA (R71D/D81R)	ComEA (R71D/D82R)
periplasmic localization	+	+	+	+	+	+	+	+	+
uniformly distributed	+	+	+	+	+	+	+	+	+
foci formation + DNA	+	+	+	+	+ (red.)	+ (red.)	-	-	-

↓ feature strain* →	ComEA (E72R)	ComEA (H79A)	ComEA (T78A/H79A)	ComEA (D81A)	ComEA (D81R)	ComEA (D82K)	ComEA (K87A)	ComEA (E91A)	ComEA (E91R)
periplasmic localization	+	+	+	+	+	+	+	+	+
uniformly distributed	+	+	+	+	+	+	+	+	+
foci formation + DNA	+	+	+	+	+	+	+	+	+

** all translationally fused to mCherry;*
# ComEA variant showed aggregation in the absence of tDNA; aggregation did not increase upon addition of DNA;
red., reduced ComEA foci observed;

B

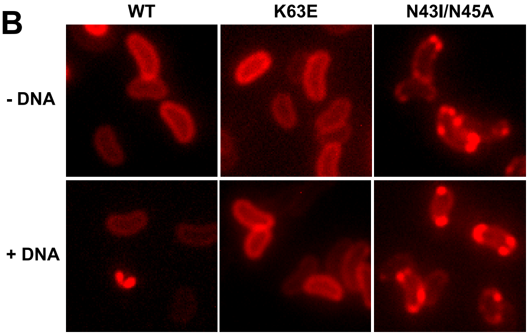

C

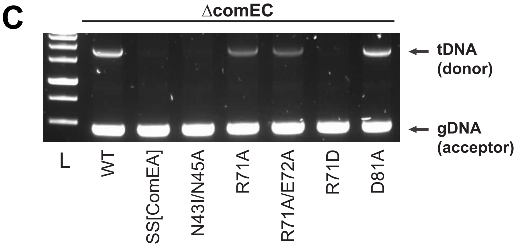

D

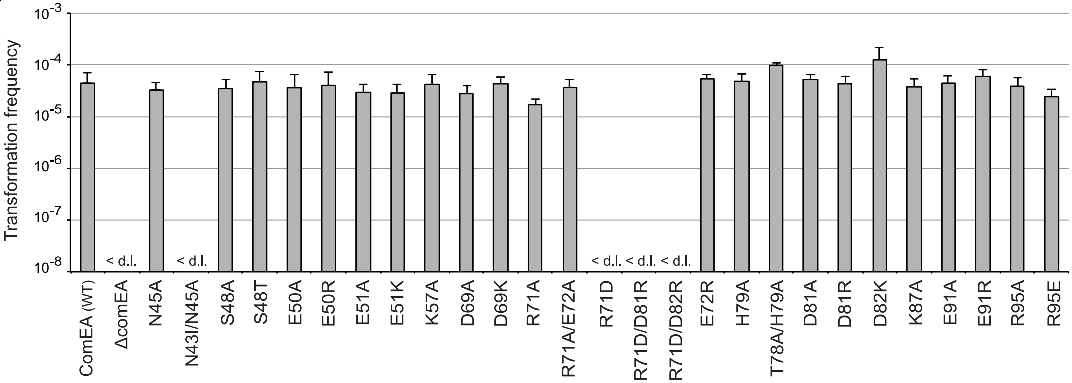

Fig. S7
