# Supplementary material for: ComEA Is Essential for the Transfer of External DNA into the Periplasm in Naturally Transformable Vibrio cholerae Cells
Source: PLoS Genet. 2014 Jan 2;10(1):e1004066. doi: 10.1371/journal.pgen.1004066 (PMC3879209; doi:10.1371/journal.pgen.1004066)
Supplement: Table S1 — Bacterial strains and plasmids. (DOCX) [file pgen.1004066.s015.docx]

| **Strain** | **Genotype^#^** | **Reference** |
| --- | --- | --- |
| ***V. cholerae*** |  |  |
| A1552 | Wild type, O1 El Tor Inaba; Rif^R^ | [[53](#_ENREF_52)] |
| A1552-Tn*tfoX* | A1552 containing mini-Tn7-*araC*-P*_BAD_*-*tfoX*; Rif^R^, Gent^R^ | [[25](#_ENREF_25)] |
| A1552-lacZ-Kan | A1552 strain with *aph* cassette in *lacZ* gene; Rif^R^, Kan^R^ | [[58](#_ENREF_57)] |
| ΔcomEC-Tn*tfoX* | A1552ΔVC1879-Tn*tfoX*; Rif^R^, Gent^R^ | [[11](#_ENREF_11)] |
| ΔcomEC::FRT-Kan-FRT-Tn*tfoX* | A1552ΔVC1879::FRT-Kan-FRT-Tn*tfoX*; Kan^R^, Rif^R^, Gent^R^ | This study |
| ΔpilA-Tn*tfoX* | A1552ΔVC2423-Tn*tfoX*; Rif^R^, Gent^R^ | [[11](#_ENREF_11)] |
| ΔcomEA-Tn*tfoX* | A1552ΔVC1917-Tn*tfoX*; Rif^R^, Gent^R^ | [[71](#_ENREF_70)] |
| ΔcomEAΔcomEC-Tn*tfoX* | A1552ΔVC1917ΔVC1879-Tn*tfoX*; Rif^R^, Gent^R^ | [[11](#_ENREF_11)] |
| ΔcomEAΔcomEC::FRT-Kan-FRT-Tn*tfoX* | A1552ΔVC1917ΔVC1879::FRT-Kan-FRT-Tn*tfoX*; Kan^R^, Rif^R^, Gent^R^ | This study |
| ΔdprA | A1552ΔVC0048; Rif^R^ | [[24](#_ENREF_24)] |
| ΔdprA-Tn*tfoX* | A1552ΔVC0048-Tn*tfoX*; Rif^R^, Gent^R^ | This study |
| ΔdprAΔcomEC-Tn*tfoX* | A1552ΔVC0048ΔVC1879-Tn*tfoX*; Rif^R^, Gent^R^ | This study |
| ComEA-mCherry-Tn*tfoX* | A1552-*comEA*-mCherry::FRT-Tn*tfoX;* Rif^R^, Gent^R^ | This study |
| ComEA-mCherry-ΔcomEC-Tn*tfoX* | A1552- *comEA*-mCherry::FRTΔVC1879-Tn*tfoX;* Rif^R^, Gent^R^ | This study |
| ComEA-mCherry-ΔpilQ-Tn*tfoX* | A1552- *comEA*-mCherry::FRTΔVC2630-Tn*tfoX;* Rif^R^, Gent^R^ | This study |
| ΔpilQ-Tn*tfoX* | A1552ΔVC2630-Tn*tfoX*; Rif^R^, Gent^R^ | [[11](#_ENREF_11)] |
| ΔpilQΔcomEC-Tn*tfoX* | A1552ΔVC2630ΔVC1879-Tn*tfoX*; Rif^R^, Gent^R^ | [[11](#_ENREF_11)] |
| ss[ComEA]-mCherry-Tn*tfoX* | A1552-Tn*tfoX* carrying gene *ss*[ComEA]*-mCherry* encoding translational fusion between signal sequence of ComEA and mCherry; replacing *comEA* (*VC1917*); Rif^R^, Gent^R^ | This study |
| ComEA^N45A^-mCherry-Tn*tfoX* | A1552-Tn*tfoX* with *comEA*[N45A]-mCherry::FRT replacing *comEA*; Rif^R^, Gent^R^ | This study |
| ComEA^N43I/N45A^-mCherry-Tn*tfoX* | A1552-Tn*tfoX* with *comEA*[N43I/N45A]-mCherry::FRT replacing *comEA*; Rif^R^, Gent^R^ | This study |
| ComEA^S48A^-mCherry-Tn*tfoX* | A1552-Tn*tfoX* with *comEA*[S48A]-mCherry::FRT replacing *comEA*; Rif^R^, Gent^R^ | This study |
| ComEA^S48T^-mCherry-Tn*tfoX* | A1552-Tn*tfoX* with *comEA*[S48T]-mCherry::FRT replacing *comEA*; Rif^R^, Gent^R^ | This study |
| ComEA^E50A^-mCherry-Tn*tfoX* | A1552-Tn*tfoX* with *comEA*[E50A]-mCherry::FRT replacing *comEA*; Rif^R^, Gent^R^ | This study |
| ComEA^E50R^-mCherry-Tn*tfoX* | A1552-Tn*tfoX* with *comEA*[E50R]-mCherry::FRT replacing *comEA*; Rif^R^, Gent^R^ | This study |
| ComEA^E51A^-mCherry-Tn*tfoX* | A1552-Tn*tfoX* with *comEA*[E51A]-mCherry::FRT replacing *comEA*; Rif^R^, Gent^R^ | This study |
| ComEA^E51K^-mCherry-Tn*tfoX* | A1552-Tn*tfoX* with *comEA*[E51K]-mCherry::FRT replacing *comEA*; Rif^R^, Gent^R^ | This study |
| ComEA^K57A^-mCherry-Tn*tfoX* | A1552-Tn*tfoX* with *comEA*[K57A]-mCherry::FRT replacing *comEA*; Rif^R^, Gent^R^ | This study |
| ComEA^G60A^-mCherry-Tn*tfoX* | A1552-Tn*tfoX* with *comEA*[G60A]-mCherry::FRT replacing *comEA*; Rif^R^, Gent^R^ | This study |
| ComEA^G60E^-mCherry-Tn*tfoX* | A1552-Tn*tfoX* with *comEA*[G60E]-mCherry::FRT replacing *comEA*; Rif^R^, Gent^R^ | This study |
| ComEA^G60V^-mCherry-Tn*tfoX* | A1552-Tn*tfoX* with *comEA*[G60V]-mCherry::FRT replacing *comEA*; Rif^R^, Gent^R^ | This study |
| ComEA^K62A^-mCherry-Tn*tfoX* | A1552-Tn*tfoX* with *comEA*[K62A]-mCherry::FRT replacing *comEA*; Rif^R^, Gent^R^ | This study |
| ComEA^K63A^-mCherry-Tn*tfoX* | A1552-Tn*tfoX* with *comEA*[K63A]-mCherry::FRT replacing *comEA*; Rif^R^, Gent^R^ | This study |
| ComEA^K63E^-mCherry-Tn*tfoX* | A1552-Tn*tfoX* with *comEA*[K63E]-mCherry::FRT replacing *comEA*; Rif^R^, Gent^R^ | This study |
| ComEA^K62/63A^-mCherry-Tn*tfoX* | A1552-Tn*tfoX* with *comEA*[K62/63A]-mCherry::FRT replacing *comEA*; Rif^R^, Gent^R^ | This study |
| ComEA^D69A^-mCherry-Tn*tfoX* | A1552-Tn*tfoX* with *comEA*[D69A]-mCherry::FRT replacing *comEA*; Rif^R^, Gent^R^ | This study |
| ComEA^D69K^-mCherry-Tn*tfoX* | A1552-Tn*tfoX* with *comEA*[D69K]-mCherry::FRT replacing *comEA*; Rif^R^, Gent^R^ | This study |
| ComEA^R71A^-mCherry-Tn*tfoX* | A1552-Tn*tfoX* with *comEA*[R71A]-mCherry::FRT replacing *comEA*; Rif^R^, Gent^R^ | This study |
| ComEA^R71D^-mCherry-Tn*tfoX* | A1552-Tn*tfoX* with *comEA*[R71D]-mCherry::FRT replacing *comEA*; Rif^R^, Gent^R^ | This study |
| ComEA^R71A/E72A^  -mCherry-Tn*tfoX* | A1552-Tn*tfoX* with *comEA*[R71A/E72A]-mCherry::FRT replacing *comEA*; Rif^R^, Gent^R^ | This study |
| ComEA^R71D/D81R^  -mCherry-Tn*tfoX* | A1552-Tn*tfoX* with *comEA*[R71D/D81A]-mCherry::FRT replacing *comEA*; Rif^R^, Gent^R^ | This study |
| ComEA^R71D/D82R^  -mCherry-Tn*tfoX* | A1552-Tn*tfoX* with *comEA*[R71D/D81R]-mCherry::FRT replacing *comEA*; Rif^R^, Gent^R^ | This study |
| ComEA^E72R^-mCherry-Tn*tfoX* | A1552-Tn*tfoX* with *comEA*[E72R]-mCherry::FRT replacing *comEA*; Rif^R^, Gent^R^ | This study |
| ComEA^H79A^-mCherry-Tn*tfoX* | A1552-Tn*tfoX* with *comEA*[H79A]-mCherry::FRT replacing *comEA*; Rif^R^, Gent^R^ | This study |
| ComEA^T78A/H79A^-mCherry-Tn*tfoX* | A1552-Tn*tfoX* with *comEA*[T78A/H79A]-mCherry::FRT replacing *comEA*; Rif^R^, Gent^R^ | This study |
| ComEA^D81A^-mCherry-Tn*tfoX* | A1552-Tn*tfoX* with *comEA*[D81A]-mCherry::FRT replacing *comEA*; Rif^R^, Gent^R^ | This study |
| ComEA^D81R^-mCherry-Tn*tfoX* | A1552-Tn*tfoX* with *comEA*[D81R]-mCherry::FRT replacing *comEA*; Rif^R^, Gent^R^ | This study |
| ComEA^D82K^-mCherry-Tn*tfoX* | A1552-Tn*tfoX* with *comEA*[D82K]-mCherry::FRT replacing *comEA*; Rif^R^, Gent^R^ | This study |
| ComEA^K87A^-mCherry-Tn*tfoX* | A1552-Tn*tfoX* with *comEA*[K87A]-mCherry::FRT replacing *comEA*; Rif^R^, Gent^R^ | This study |
| ComEA^E91A^-mCherry-Tn*tfoX* | A1552-Tn*tfoX* with *comEA*[E91A]-mCherry::FRT replacing *comEA*; Rif^R^, Gent^R^ | This study |
| ComEA^E91R^-mCherry-Tn*tfoX* | A1552-Tn*tfoX* with *comEA*[E91R]-mCherry::FRT replacing *comEA*; Rif^R^, Gent^R^ | This study |
| ComEA^E91S^-mCherry-Tn*tfoX* | A1552-Tn*tfoX* with *comEA*[E91S]-mCherry::FRT replacing *comEA*; Rif^R^, Gent^R^ | This study |
| ComEA^R95A^-mCherry-Tn*tfoX* | A1552-Tn*tfoX* with *comEA*[R95A]-mCherry::FRT replacing *comEA*; Rif^R^, Gent^R^ | This study |
| ComEA^R95E^-mCherry-Tn*tfoX* | A1552-Tn*tfoX* with *comEA*[R95E]-mCherry::FRT replacing *comEA*; Rif^R^, Gent^R^ | This study |
| ΔcomEC::FRT-kan-FRT-Tn*tfoX* | A1552ΔVC1879::FRT-Kan-FRT-Tn*tfoX*; Rif^R^, Kan^R^, Gent^R^ | This study |
| ComEA-mCherry ΔcomEC::FRT-kan-FRT-Tn*tfoX* | ComEA-mCherry ΔVC1879::FRT-Kan-FRT-Tn*tfoX*; Rif^R^, Kan^R^, Gent^R^ | This study |
| ss[ComEA]-mCherry ΔcomEC::FRT-kan-FRT-Tn*tfoX* | ss[ComEA]-mCherry ΔVC1879::FRT-Kan-FRT-Tn*tfoX*; Rif^R^, Kan^R^, Gent^R^ | This study |
| ComEA^G60A^-mCherry ΔcomEC::FRT-kan-FRT-Tn*tfoX* | ComEA^G60A^-mCherry ΔVC1879::FRT-Kan-FRT-Tn*tfoX*; Rif^R^, Kan^R^, Gent^R^ | This study |
| ComEA^G60V^-mCherry ΔcomEC::FRT-kan-FRT-Tn*tfoX* | ComEA^G60V^-mCherry ΔVC1879::FRT-Kan-FRT-Tn*tfoX*; Rif^R^, Kan^R^, Gent^R^ | This study |
| ComEA^G60E^-mCherry ΔcomEC::FRT-kan-FRT-Tn*tfoX* | ComEA^G60E^-mCherry ΔVC1879::FRT-Kan-FRT-Tn*tfoX*; Rif^R^, Kan^R^, Gent^R^ | This study |
| ComEA^K62A^-mCherry ΔcomEC::FRT-kan-FRT-Tn*tfoX* | ComEA^K62A^-mCherry ΔVC1879::FRT-Kan-FRT-Tn*tfoX*; Rif^R^, Kan^R^, Gent^R^ | This study |
| ComEA^K63A^-mCherry ΔcomEC::FRT-kan-FRT-Tn*tfoX* | ComEA^K63A^-mCherry ΔVC1879::FRT-Kan-FRT-Tn*tfoX*; Rif^R^, Kan^R^, Gent^R^ | This study |
| ComEA^K63E^-mCherry ΔcomEC::FRT-kan-FRT-Tn*tfoX* | ComEA^K63E^-mCherry ΔVC1879::FRT-Kan-FRT-Tn*tfoX*; Rif^R^, Kan^R^, Gent^R^ | This study |
| ComEA^K62/63A^-mCherry ΔcomEC::FRT-kan-FRT-Tn*tfoX* | ComEA^K62/63A^-mCherry ΔVC1879::FRT-Kan-FRT-Tn*tfoX*; Rif^R^, Kan^R^, Gent^R^ | This study |
| ComEA^N43I/N45A^-mCherry  ΔcomEC::FRT-kan-FRT-Tn*tfoX* | ComEA^N43I/N45A^-mCherry ΔVC1879::FRT-Kan-FRT-Tn*tfoX*; Rif^R^, Kan^R^, Gent^R^ | This study |
| ComEA^R71A^-mCherry ΔcomEC::FRT-kan-FRT-Tn*tfoX* | ComEA^R71A^-mCherry ΔVC1879::FRT-Kan-FRT-Tn*tfoX*; Rif^R^, Kan^R^, Gent^R^ | This study |
| ComEA^R71A/E72A^-mCherry ΔcomEC::FRT-kan-FRT-Tn*tfoX* | ComEA^R71A/E72A^-mCherry ΔVC1879::FRT-Kan-FRT-Tn*tfoX*; Rif^R^, Kan^R^, Gent^R^ | This study |
| ComEA^R71D^-mCherry ΔcomEC::FRT-kan-FRT-Tn*tfoX* | ComEA^R71D^-mCherry ΔVC1879::FRT-Kan-FRT-Tn*tfoX*; Rif^R^, Kan^R^, Gent^R^ | This study |
| ComEA^D81A^-mCherry ΔcomEC::FRT-kan-FRT-Tn*tfoX* | ComEA^D81A^-mCherry ΔVC1879::FRT-Kan-FRT-Tn*tfoX*; Rif^R^, Kan^R^, Gent^R^ | This study |
| ComEAΔHhH1-mCherry-Tn*tfoX* | A1552-Tn*tfoX* with *comEA*ΔHhH1-mCherry::FRT replacing *comEA*; Rif^R^, Gent^R^ | This study |
| ComEAΔHhH2-mCherry-Tn*tfoX* | A1552-Tn*tfoX* with *comEA*ΔHhH2-mCherry::FRT replacing *comEA*; Rif^R^, Gent^R^ | This study |
| ComEA-*B.s.*-mCherry-Tn*tfoX* | A1552-Tn*tfoX* with *comEA* from *B. subtilis (B.s.)-*mCherry::FRT; transmembrane domain of ComEA*^B.s.^* removed and replaced by signal sequence of ComEA*^V.c.^*; replacing *comEA*; Rif^R^, Gent^R^ | This study |
| ComEA-*B.s.-*Tn*tfoX* | A1552-Tn*tfoX* with *comEA* from *B. subtilis (B.s.)*::FRT; transmembrane domain of ComEA*^B.s.^* removed and replaced by signal sequence of ComEA*^V.c.^*; replacing *comEA*; Rif^R^, Gent^R^ | This study |
| ComEA (C-term)-*B.s.*-mCherry-Tn*tfoX* | A1552-Tn*tfoX* encoding C-terminal part of ComEA from *B. subtilis (B.s.)*-mCherry::FRT; fused to signal sequence of ComEA*^V.c.^*; replacing *comEA*; Rif^R^, Gent^R^ | This study |
| ComEA (C-term)-*B.s.*- Tn*tfoX* | A1552-Tn*tfoX* encoding C-terminal part of ComEA from *B. subtilis (B.s.)*::FRT; fused to signal sequence of ComEA*^V.c.^*; replacing *comEA*; Rif^R^, Gent^R^ | This study |
| ComE1-*N.g.*-mCherry-Tn*tfoX* | A1552-Tn*tfoX* with *comE1* from *N. gonorrhoeae (N.g.)-*mCherry::FRT; signal sequence of ComE1^N.g.^ removed and replaced by signal sequence of ComEA*^V.c.^*; replacing *comEA*; Rif^R^, Gent^R^ | This study |
| ComE1-*H.i.*-mCherry-Tn*tfoX* | A1552-Tn*tfoX* with *comE1* from *H. influenzae (H.i.)-*mCherry::FRT; signal sequence of ComE1^H.i.^ removed and replaced by signal sequence of ComEA*^V.c.^*; replacing *comEA*; Rif^R^, Gent^R^ | This study |
| ComE1-*P.m.*-mCherry-Tn*tfoX* | A1552-Tn*tfoX* with *comE1* from *P. multocida (P.m.)-*mCherry::FRT; signal sequence of ComE1^P.m.^ removed and replaced by signal sequence of ComEA*^V.c.^*; replacing *comEA*; Rif^R^, Gent^R^ | This study |
| ComEA-bla-Tn*tfoX* | A1552-*comEA*-*bla*::FRT-Tn*tfoX;* Rif^R^, Gent^R,^ Amp^R^ | This study |
| Δdns-Tn*tfoX* | A1552-ΔVC0470-Tn*tfoX*; Rif^R^, Gent^R^ | This study |
| Δxds-Tn*tfoX* | A1552-ΔVC2621-Tn*tfoX*; Rif^R^, Gent^R^ | This study |
| ΔdnsΔxds-Tn*tfoX* | A1552-ΔVC0470ΔVC2621-Tn*tfoX*; Rif^R^, Gent^R^ | This study |
| ΔdnsΔxdsΔcomEC::FRT-Kan-FRT-Tn*tfoX* | A1552-ΔVC0470ΔVC2621ΔVC1879::FRT-Kan-FRT-Tn*tfoX*; Kan^R^, Rif^R^, Gent^R^ | This study |
| ΔdnsΔxdsΔcomEA-Tn*tfoX* | A1552-ΔVC0470ΔVC2621ΔVC1917-Tn*tfoX*; Rif^R^, Gent^R^ | This study |
| ΔdnsΔxdsΔcomEAΔcomEC::FRT-Kan-FRT-Tn*tfoX* | A1552-ΔVC0470ΔVC2621ΔVC1917ΔVC1879::FRT-Kan-FRT-Tn*tfoX*; Kan^R^, Rif^R^, Gent^R^ | This study |
|  |  |  |
| **Plasmid** | **Description** | **Reference** |
| pBAD/Myc-HisA | pBR322-derived expression vector; *araBAD* promoter (P_BAD_); Amp^R^ | Invitrogen |
| pUX-BF13 | *ori*R6K, helper plasmid with Tn7 transposition function; Amp^R^ | [[77](#_ENREF_76)] |
| pBR-flp | FLP^+^, **λ** cI857^+^, **λ** *p*_R_ Rep^ts^ integrated into *Eco*RV site of pBR322 | [[55](#_ENREF_54)] |
| pGP704-mTn7-*araC*-*tfoX* | pGP704 with mini-Tn7 carrying *araC* and P_BAD_-driven *tfoX*; Amp^R^ | [[25](#_ENREF_25)] |
| pBAD-*comEA* | *comEA* gene cloned into pBAD/Myc-HisA, arabinose inducible; Amp^R^ | [[71](#_ENREF_70)] |
| pBAD(kan) | *bla* replaced by *aph* in pBAD/Myc-HisA; promoter region and MCS maintained; Kan^R^ | This study |
| pBAD(kan)-*comEA* | *comEA* gene cloned into pBAD(kan), arabinose inducible; Kan^R^ | This study |
| pBAD(kan)-*comEA-*mCherry | *comEA-mCherry* gene cloned into pBAD(kan), arabinose inducible; Kan^R^ | This study |
| pBAD(kan)*-*ss[ComEA]*-*mCherry | *ss*[ComEA]*-mCherry* gene cloned into pBAD(kan), arabinose inducible; Kan^R^ | This study |
| pBAD*-*tat-GFP | Tat-dependent signal sequence of *torA* (VC1692) translationally fused to *gfp*; cloned into pBAD/Myc-HisA; arabinose inducible; Amp^R^ | This study |
| pBAD*-*tat-*comEA-*GFP | Tat-dependent signal sequence of *torA* (VC1692) translationally fused to *comEA* (lacking native and sec-dependent signal sequence); cloned into pBAD/Myc-HisA; arabinose inducible; Amp^R^ | This study |
| pBAD-tat-*comEA*^K63A^  -GFP | Derivative of pBAD-tat-*comEA*-GFP; WT *comEA* was changed to *comEA*[K63A]; arabinose inducible; Amp^R^ | This study |
| pBAD-tat-*comEA*^K63E^  -GFP | Derivative of pBAD-tat-*comEA*-GFP; WT *comEA* was changed to *comEA*[K63E]; arabinose inducible; Amp^R^ | This study |
| pBAD-tat-*comEA*^K62/63A^  -GFP | Derivative of pBAD-tat-*comEA*-GFP; WT *comEA* was changed to *comEA*[K62/63A]; arabinose inducible; Amp^R^ | This study |
| pBAD-tat-*comEA*^ΔHhH1^  -GFP | Derivative of pBAD-tat-*comEA*-GFP; WT *comEA* was changed to *comEA*[ΔHhH1]; arabinose inducible; Amp^R^ | This study |
| pBAD-tat-*comEA*^ΔHhH2^  -GFP | Derivative of pBAD-tat-*comEA*-GFP; WT *comEA* was changed to *comEA*[ΔHhH2]; arabinose inducible; Amp^R^ | This study |
| pBAD-*comEA-*strep | Strep-tagII translationally fused to *comEA* using inverse PCR on plasmid pBAD-*comEA*; Amp^R^ | This study |
| pBAD-*comEA*^K62/63A^  *-*strep | *comEA-*strep site-directly modified by inverse PCR to exchange K62 and K63 by alanine residues in the encoded protein; template used: pBAD-*comEA*-strep; Amp^R^ | This study |
| pBAD(kan)-*comEA-*mCherry-strep | Strep-tagII translationally fused to *comEA* using inverse PCR on plasmid pBAD(kan)-*comEA-*mCherry; Kan^R^ | This study |
| pBAD(kan)- *comEA*^K62/63A^- mCherry*-*strep | *comEA-*strep site-directly modified by inverse PCR to exchange K62 and K63 by alanine residues in the encoded protein; template used: pBAD(kan)-*comEA-*mCherry-strep; Kan^R^ | This study |

^#^ VC numbers according to Ref [[78](#_ENREF_77)]

77. Bao Y, Lies DP, Fu H, Roberts GP (1991) An improved Tn*7*-based system for the single-copy insertion of cloned genes into chromosomes of gram-negative bacteria. Gene 109: 167-168.

78. Heidelberg JF, Eisen JA, Nelson WC, Clayton RA, Gwinn ML, et al. (2000) DNA sequence of both chromosomes of the cholera pathogen *Vibrio cholerae*. Nature 406: 477-483.
