# Supplementary material for: ComEA Is Essential for the Transfer of External DNA into the Periplasm in Naturally Transformable Vibrio cholerae Cells
Source: PLoS Genet. 2014 Jan 2;10(1):e1004066. doi: 10.1371/journal.pgen.1004066 (PMC3879209; doi:10.1371/journal.pgen.1004066)
Supplement: Figure S6 — The absence of the nucleases Dns and Xds does not rescue the ΔcomEA phenotype. (A) Natural transformation of the indicated strains was scored using a chitin-independent transformation assay [25]. The transformation frequencies shown on the Y-axis are averaged from at least three independent replicates (± SD). <d.l., below detection limit. Statistically significant differences are indicated (**P<0.01); n.s. not statistically different. (B) DNA uptake assay as described for Fig. 2. The genotypes of the tested strains are indicated above the figure. L, ladder. (PDF) [file pgen.1004066.s006.pdf]

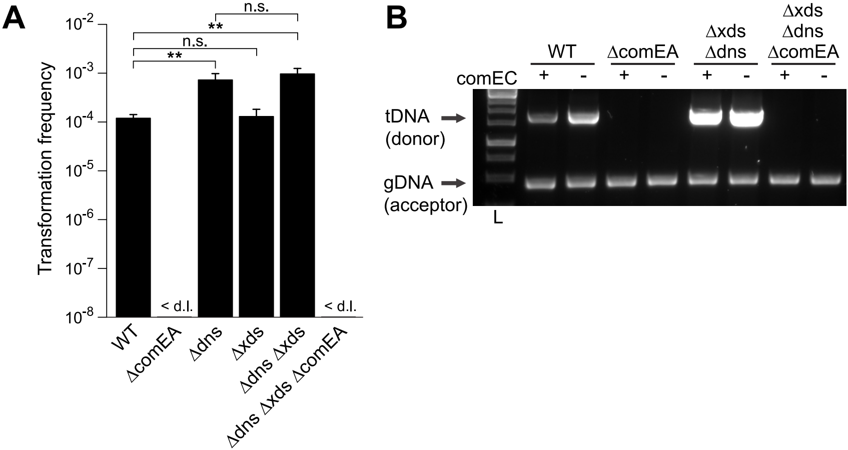
A
Transformation frequency
10-2
10-3
10-4
10-5
10-6
10-7
10-8
n.s.
**
n.s.
**
< d.l.
< d.l.
WT
ΔcomEA
Δdns
Δxds
Δdns Δxds
Δdns Δxds ΔcomEA
B
WT
ΔcomEA
Δxds Δdns
Δxds Δdns ΔcomEA
comEC
+ - + - + - + -
tDNA (donor)
gDNA (acceptor)
L

Fig. S6
